# Ecdysterone Alleviates Atherosclerosis by Inhibiting NCF2 and Inhibiting Ferroptosis Mediated by the PI3K/Akt/Nrf2 Pathway

**DOI:** 10.1111/jcmm.70446

**Published:** 2025-03-05

**Authors:** Zhenyu Wang, Fengchao Wu, Ju Yan, Lei Liang, Fengjun Chang, Mengya Dong, Jiayu Diao, Haoyu Wu

**Affiliations:** ^1^ Department of Cardiology Shaanxi Provincial People's Hospital Xi'an Shaanxi China

**Keywords:** atherosclerosis, ecdysterone, ferroptosis, NCF2, pI3K/Akt/Nrf2 pathway

## Abstract

Ecdysterone (Ecd), an active ingredient in trianthema, has a strong anti‐inflammatory effect. This study aimed to explore the potential mechanism by which Ecd improves atherosclerosis (AS). Here, we systematically investigated the mechanism of Ecd in human umbilical vein endothelial cells (HUVECs) treated with oxidised low‐density lipoprotein (ox‐LDL). In ox‐LDL‐treated HUVECs, Ecd promoted HUVEC viability as well as inhibited ferroptosis and the secretion of inflammatory factors (TNF‐α, IL‐6 and IL‐1β). In addition, Ecd inhibited the expression of neutrophil cytoplasmic factor 2 (NCF2) and triggered the PI3K/AKT/Nrf2 signalling pathway, thereby alleviating the increase of ferroptosis in ox‐LDL‐treated HUVECs. More importantly, we constructed an AS mouse model by feeding ApoE^−/−^ mice with a high‐fat diet and found that Ecd treatment alleviated vasculopathy and arterial ferroptosis and inhibited the secretion of inflammatory factors in vivo, which could be reversed by overexpression of NCF2. Overall, this study showed that the protective effect of Ecd on AS is mainly achieved by inhibiting NCF2 and activating the PI3K/Akt/Nrf2 pathway to inhibit ferroptosis. Therefore, Ecd may be an effective drug to improve AS by inhibiting ferroptosis‐induced inflammation.

## 1 Introduction

Atherosclerosis (AS) is a chronic inflammatory vascular disease [1], which mainly manifests as lipid accumulation, plaque rupture and thrombosis of the vascular wall [2]. A variety of risk factors and molecular mechanisms, such as hyperlipidemia, hyperglycemia and local or systemic inflammatory responses, can induce AS [3]. Furthermore, AS also induces a variety of diseases such as coronary artery disease, stroke and cerebrovascular disease [4]. At present, AS treatment is still dominated by drug therapy (including statins, antiplatelet drugs and vasodilators). However, owing to the non‐specific distribution and poor water solubility of drugs, there are some limitations in the treatment of AS, which may lead to side effects such as liver injury, muscle pain and diabetes [5]. Therefore, there is an urgent need to explore the molecular mechanisms of AS pathogenesis and identify promising drugs for the prevention of AS.

Ferroptosis is a new type of cell death caused by iron‐dependent lipid peroxidation [6]. After ferroptosis, intracellular glutathione (GSH) is depleted, and GSH peroxidase 4 (Gpx4) activity is reduced, which is usually accompanied by a large amount of iron accumulation and lipid peroxidation (ROS) [7, 8, 9]. Many diseases have been shown to be associated with ferroptosis, including cancer [10], Alzheimer disease [11], psoriasis [12] and inflammation‐related diseases [13]. A study has found that the increase of ferroptosis is associated with the occurrence of AS [14], and inhibition of ferroptosis can alleviate AS by reducing lipid peroxidation [15]. In addition, the PI3K/Akt/Nrf2 pathway can affect ferroptosis, and activation of the PI3K/Akt/Nrf2 pathway can reduce ferroptosis [16]. Therefore, it is important to explore the biological pathways related to the changes in the PI3K/Akt/Nrf2 pathway and ferroptosis in the progression of AS for a comprehensive understanding of the pathogenesis of AS.

Ecdysterone (Ecd) is the main component of *Trianthema portulacastrum* [17], alias 20‐Ecd, β‐Ecd, and is a natural active steroid for insect moulting. Ecd is closely related to a variety of pathological mechanisms and physiological phenomena, including apoptosis, autophagy, oxidative stress, tumour growth and immune response [18, 19, 20]. A study found that Ecd can reduce osteoporosis in mice [21]. Studies have also reported that Ecd can attenuate cardiac remodelling in spontaneously hypertensive rats and inhibit the inflammatory response [22, 23]. An early study also demonstrated that Ecd influences experimental AS in rabbits [24]. In addition, Ecd induces expression by activating the PI3K/Akt/Nrf2 signalling pathway, thereby protecting PC12 cells from MPP^+^‐induced neurotoxicity [25]. Therefore, a comprehensive and systematic understanding of the mechanism of action of Ecd in AS may provide a theoretical basis for AS treatment.

Neutrophil cytoplasmic factor 2 (NCF2) is a subunit of NADPH oxidase [26], which is involved in the production of ROS [27]. Previous studies have demonstrated that NCF2 can be used as a key gene for the diagnosis of AS using bioinformatics tools [28, 29]. NCF2 is also closely related to oxidative stress, autophagy, apoptosis and ferroptosis [30, 31]. The study found that cells incubated with 3H‐labelled steroids in the presence of NADPH produced 20,26‐dihydroxyecdysone, which can be used as a product of Ecd [26], and both Ecd and NCF2 can affect the inflammatory response. A recent study also found that NCF2 can be used as a ferroptosis‐related gene and is significantly enriched in the PI3k/Akt pathway [32]. NCF2 is also involved in the regulation of the Nrf2 pathway [33]. Therefore, we speculate that Ecd is associated with NCF2 in AS and is involved in the regulation of the PI3k/Akt/Nrf2 pathway and ferroptosis.

Based on the literature, this study screened the active ingredient Ecd of *T. portulacastrum* and explored its role in AS and its possible molecular mechanism, providing new potential therapeutic targets for its application and development.

## 2 Materials and Methods

### 2.1 Cell Culture and Oxidised Low‐Density Lipoprotein (Ox‐LDL) Treatment

Human umbilical vein endothelial cells (HUVECs) were purchased from the Shanghai Cell Bank of the Chinese Academy of Sciences (SCSP‐5285, Shanghai, China). HUVECs were cultured in DMEM (11320033, Thermo Fisher Scientific, Boston, MA, USA) containing 10% fetal bovine serum (FBS) and 100 U/mL penicillin in a humidified atmosphere containing 5% CO_2_ at 37°C.

HUVECs were treated with 100 μg/mL ox‐LDL (L8292, Sigma‐Aldrich, St. Louis, MO, USA) for 24 h to induce cell injury. Ecd (purity: 98%) was purchased from Shanghai Dingrui Chemical Co. Ltd. (DR11005, Shanghai, China) and was dissolved in ethanol. HUVECs were treated in the absence or presence of 10, 20, 40, 80, 120 or 160 μM Ecd for 24 h after ox‐LDL treatment. Ethanol without Ecd was used as a control.

### 2.2 Cell Transfection

pcDNA‐NCF2 and pcDNA3.1 were purchased from Thermo Fisher Scientific. A small interfering RNA against NCF2 (si‐NCF2) was designed, synthesised and validated by Han Heng Biology (Shanghai, China). Subsequently, these vectors were transfected into ox‐LDL‐treated HUVECs using the Lipofectamine 2000 reagent (11668500, Thermo Fisher Scientific). The transfected cells were cultured at 37°C with 5% CO_2_ for 48 h for subsequent in vitro experiments, according to the kit manufacturer's instructions. The NCF2 lentiviral recombinant vector was constructed and packaged at Yunzhou Biology.

### 2.3 QPCR

Total RNA was isolated using the TRIzol reagent (15596026CN, Thermo Fisher Scientific). cDNA was synthesised using the PrimeScript RT Reagent Kit (6210A, TaKaRa, Biotechnology, Dalian, China). Subsequently, TB Green Premix Ex TaqTM II (R004Q, TaKaRa) was used for qPCR to detect the levels of NCF2, TNK‐α, IL‐6 and IL‐1β mRNAs. The following steps were performed: 10 min at 95°C and 35 cycles of 15 s at 95°C, 20 s at 60°C and 15 s at 72°C. Quantification was performed using the 2^−ΔΔ*Ct*^ method after normalisation with an internal control (β‐actin) [34]. The primers used in qPCR are as follows: NCF2, forward: 5′‐ACG CTC CAA CCT GTC TTC TC‐3′; reverse: 5′‐TTC CAG TCC TTC TTG TCC GC‐3′; β‐actin, forward: 5′‐TCG TGC GTG ACA TTA AGG AG‐3′; reverse: 5′‐GTC AGG CAG CTC GTA GCT CT‐3′; TNK‐α, forward: 5′‐TGA TCG GTC CCC AAA GGG ATG‐3′; reverse: 5′‐TTG GTG GTT TGC TAC GAC GTG G‐3′; IL‐6: forward: 5′‐TGA TGC ACT TGC AGA AAA CAA TCT GA‐3′; reverse: 5′‐AGC TAT GGT ACT CCA GAA GAC CAG AGG‐3′; IL‐1β, forward: 5′‐GCA ACT GTT CCT GAA CTC AAC T‐3′; reverse: 5′‐ATC TTT TGG GGT CCG TCA ACT‐3′.

### 2.4 Western Blotting

Western blotting was performed as previously described [35]. Briefly, total protein was extracted from cells and tissues using a protein extraction kit (SD‐001/SN‐002, Invent Biotechnologies, Eden Prairie, MN, USA). Total protein concentration was determined using a BCA kit (23250, Thermo Fisher Scientific). Protein samples were separated by electrophoresis and transferred to PVDF membranes (88585, Thermo Fisher Scientific). Subsequently, the membrane was sealed at room temperature and incubated with the primary antibodies rabbit‐anti‐NCF2 (1:500, ab109523, Cambridge, Abcam, UK), rabbit‐anti‐ACSL4(1:500, ab155282, Abcam), rabbit‐anti‐p‐Akt (1:100, ab8805, Abcam), rabbit‐anti‐p‐PI3K (1:200, ab191606, Abcam), rabbit‐anti‐Gpx4 (1:200, ab125066, Abcam), rabbit‐anti‐FTH1 (1:200, ab183781, Abcam), rabbit‐anti‐Nrf2 (1:100, ab137550, Abcam), and rabbit anti‐β‐actin (1:100, 4970, Cell Signalling Technology Inc.) at 4°C overnight. Subsequently, the membranes were incubated with the secondary antibody IgG H&L (HRP) (1:200, ab6721, Abcam, UK) at room temperature for 1 h. An enhanced chemiluminescence kit (36222ES, Yisheng Biotechnology, Shanghai, China) was used to detect protein expression. ImageJ software (NIH, Bethesda, MD, USA) was used for the quantitative analysis of bands.

### 2.5 Detection of Ferroptosis‐Related Indicators

The contents of Fe^2+^, MDA and 4‐HNE, the ratio of GSH/GSSG, as well as the activities of Gpx4 and SOD were detected with the Iron Concentration Assay Kit (ab83366, Abcam), MDA detection kit (MAK085, Sigma‐Aldrich), 4‐HNE detection kit (MAK085, Abcam), the GSH and GSSG assay kit (HZ0053, HuZhen Biology, Shanghai, China), GPX analysis kit (BA1115, Shangbao Biology, China) and SOD assay kit (ab65354, Abcam), respectively.

### 2.6 Detection of Intracellular ROS

The DCFH‐DA probe (HY‐D0940, MedChemExpress, New Jersey City, NJ, USA) was added to the treated HUVECs. Cells were cultured in an incubator at 37°C for 20 min. Subsequently, HUVECs were collected, and the fluorescence intensity of HUVECs in different treatment groups was detected by FACSCalibur flow cytometry (Becton Dickinson and Company, Franklin Lakes, NJ, USA).

### 2.7 Enzyme‐Linked Immunosorbent Assay (ELISA)

The serum levels of total cholesterol (TC), triglyceride (TG), high‐density lipoprotein (HDL) and LDL were measured using commercial biochemical kits (Nanjing Jiancheng Bioengineering Institute, Nanjing, China). The level of NO was quantified using an assay kit (BH‐961193, Bohu Biotechnology, Shanghai, China) according to the manufacturer's protocol, and eNOS (ml025093, MlBio, Shanghai, China) and ET‐1 (ml981433, MlBio, Shanghai, China) activities were determined using ELISA kits.

The concentrations of TNF‐α (ab181421, Abcam), IL‐6 (ab178013, Abcam) and IL‐1β (ab214025, Abcam) were measured using ELISA kits in the culture supernatant following the manufacturer's instructions.

The concentrations of TNF‐α (ab208348, Abcam), IL‐6 (ab222503, Abcam) and IL‐1β (ab197742, Abcam) were measured using ELISA kits in the serum of mice following the manufacturer's instructions.

### 2.8 Cell Apoptosis Analysis

The apoptosis of HUVECs was evaluated using an Annexin V/FITC and propidium iodide (PI) apoptosis detection kit (550,911, Becton Dickinson and Company) according to the manufacturer's instructions. Briefly, the treated cells were collected and suspended in annexin‐binding buffer, followed by staining with Annexin V‐FITC/PI for 15 min in the dark at room temperature. Subsequently, the stained cells were analysed using a CYTOMICS FC 500 flow cytometer (Beckman Coulter, USA).

### 2.9 Drug Affinity Reaction Target Stability (DARTS) Analysis

The total protein in the cell lysate was extracted, and the lysate was equally divided into samples containing 100 μg of protein and incubated with or without Ecd at 25°C for 10 min. After that, the protease was added to continue the incubation. After the incubation was completed, the water bath was heated to terminate the protease hydrolysis of the protein. The samples were separated by SDS‐PAGE and stained to detect the stability of the target protein.

### 2.10 AS Mouse Model Construction

Sixteen male C57BL/6 wild‐type (WT) mice and 40 male ApoE^−/−^ mice on a C57BL/6 background (aged 6–8 weeks old, weighing 18–20 g) were purchased from the Xi'an JiaoTong University Laboratory Animal Center to establish mouse models of AS. The experimental mice were housed at 25°C ± 1°C under a 12 h light/12 h dark cycle, with free access to food and water. After one‐week adaptation, the mice were fed a high‐fat diet (21% fat and 0.15% cholesterol) for 10 weeks. ApoE^−/−^ mice were randomly divided into the AS, AS+Ecd and AS+Ecd + NCF2 groups. WT C57BL/6 mice were fed normally as the control group, with eight mice in each group. In addition, atorvastatin (ATO) was selected as a positive control drug to analyse its alleviation effect on AS in mice. In this experiment, ApoE^−/−^ mice were randomly divided into the AS and AS+ATO groups, and WT mice were used as the control group. This study was approved by the Ethics Committee of the Shaanxi Provincial People's Hospital. Animal experiments were performed according to the ‘Laboratory Animal Care and Use Guide’ (Approval number: SPPH‐2022‐28A).

### 2.11 Oil Red O Staining

At the end of the experiment, the aorta was stripped and fixed in neutral formaldehyde for 24 h. The aorta was then infiltrated with 60% isopropanol for 10 min and soaked in oil red O dye solution (C0157M, Beyotime, Shanghai, China) for 10 min. Excess oil red O dye was removed with 60% isopropanol, and the aorta was washed with water for 3 min. Finally, the degree of arterial plaque formation was observed, and images were collected. The size of the facial lesions was estimated by calculating the ratio of oil red O‐positive staining to the total surface area [36].

### 2.12 Haematoxylin/Eosin (H&E) Staining

The arterial tissue was immersed in 10% formalin for 30 min and dehydrated overnight in 75% ethanol. The tissue was then embedded in paraffin and cut into 4 μm sections. The sections were then subjected to histopathological examination by H&E staining kit (ab245880, Abcam).

### 2.13 Statistical Analyses

GraphPad 8.0.2 was utilised for the data analyses. Student's *t*‐test and ANOVA were applied to analyse significant differences, and Tukey's test was used for post hoc analysis of variance (ANOVA) tests. The data are expressed as mean ± standard deviation (means ± SD). *p* < 0.05 was defined as statistically significant.

## 3 Results

### 3.1 Ecd Promotes Ox‐LDL‐Treated HUVECs Viability and Inhibits Ferroptosis

**FIGURE 1:**
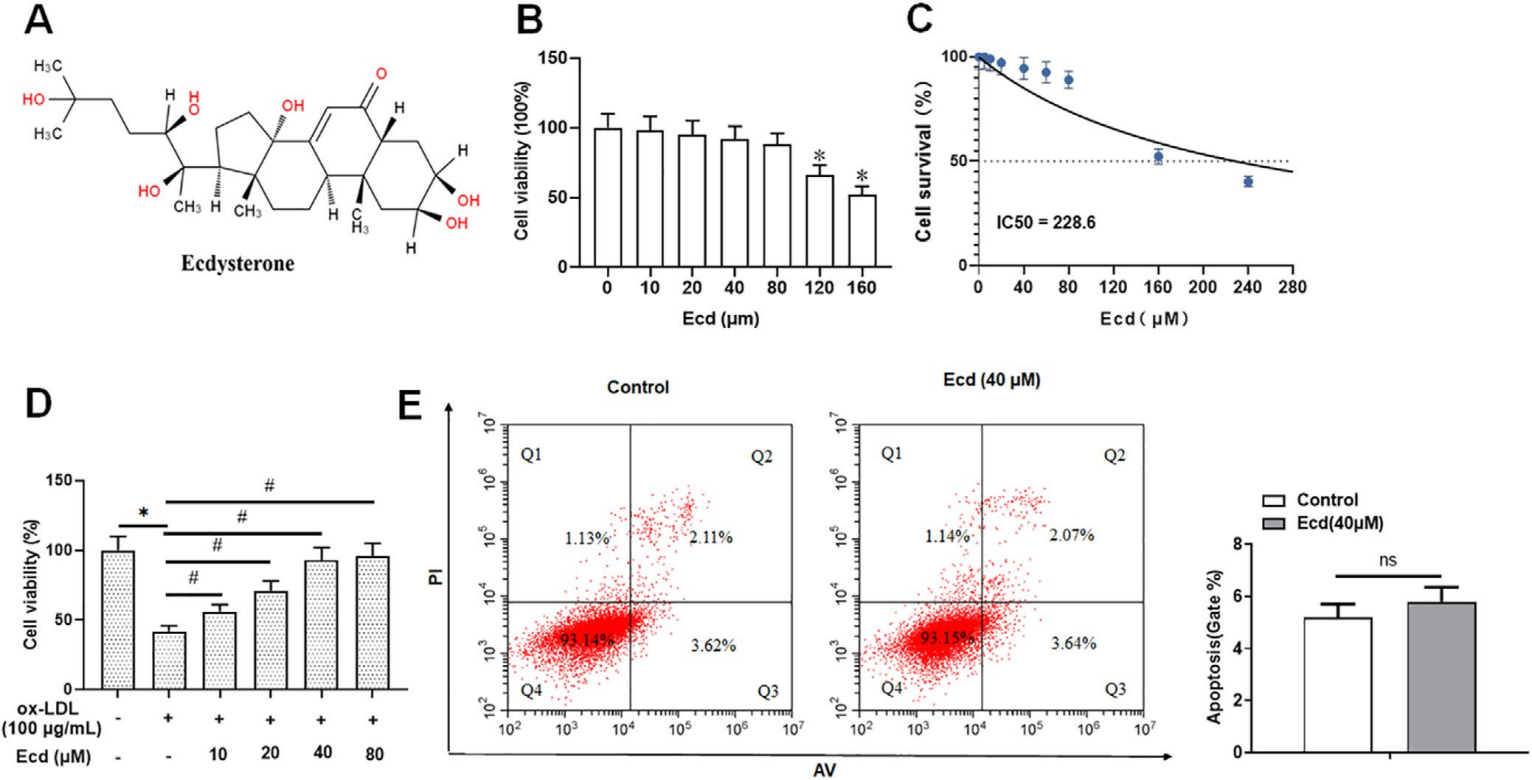
Ecd promotes ox‐LDL‐treated viability of HUVECs. (A) Chemical skeleton structure of Ecd; (B) HUVECs were treated with different concentrations of Ecd (0, 10, 20, 40, 80, 120 or 160 μM) for 24 h and cell viability was measured; (C) The EC50 value of Ecd was determined by MTT; (D) Cell viability was measured in cells treated with Ecd (10, 20, 40 or 80 μM) followed by the treatment of ox‐LDL (100 μM, 24 h); (E) The level of apoptosis was detected by flow cytometry; the data is expressed in means ± SD, *n* = 4; Student's *t* test was used for comparison between the two groups, and one‐way analysis of variance was used for comparison between multiple groups; compared with 0 μM group *p < 0.05, compared with 100 μM ox‐LDL group #*p* < 0.05.

The Ecd chemical structure is shown in Figure 1A, and the effect of Ecd on HUVECs viability is shown in Figure 1B. The results showed that Ecd decreased HUVECs viability at concentrations > 120 μM. The in vitro safety results showed that Ecd concentration less than 80 μM had no significant inhibitory effect on HUVEC activity, and the IC_50_ value was 228.6 μM (Figure 1C). HUVECs were then administered Ecd (10, 20, 40 or 80 μM) after ox‐LDL stimulation (100 μg/mL), and the results showed that Ecd administration significantly alleviated ox‐LDL‐treated HUVECs injury in a dose‐dependent manner (Figure 1D). The results of flow cytometry showed that 40 μM Ecd had no effect on HUVEC apoptosis (Figure 1E). Therefore, 40 μM Ecd is considered a safe concentration for treating HUVECs.

**FIGURE 2:**
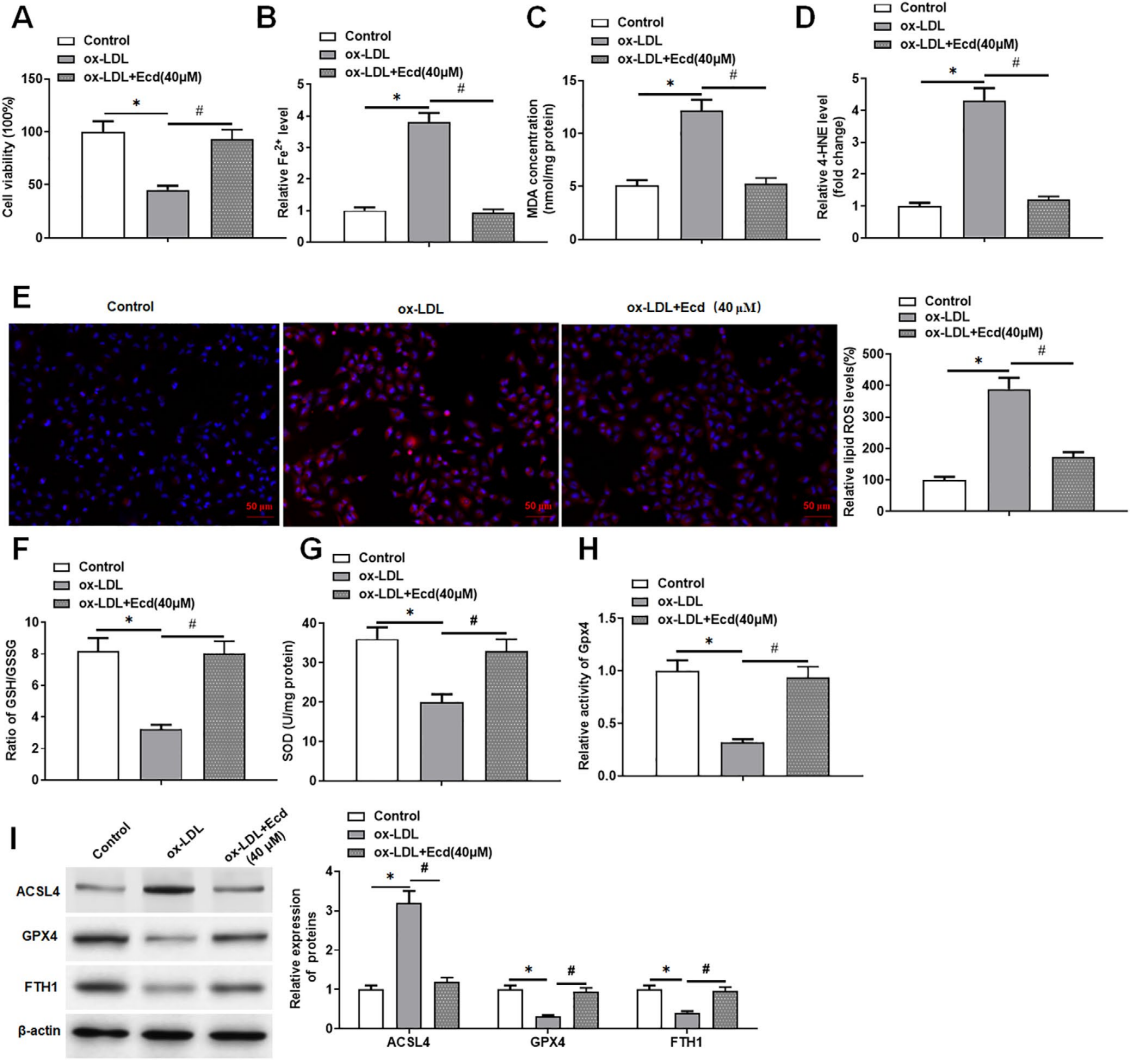
Ecd inhibits ox‐LDL‐treated ferroptosis in HUVECs. HUVECs were treated with Ecd (40 μM) for 24 h and then with 100 μM ox‐LDL for 24 h. (A) MTT assay was used to detect the viability of HUVECs; (B) Iron assay kit to detect ferrous ion levels; (C) MDA detection kit to detect MDA content; (D) 4‐HNE detection kit to evaluate the level of 4‐HNE; (E) DCFH‐DA fluorescent probe method was used to detect lipid ROS levels (original magnification, 200×) Scale bars = 1 cm; (F) GSH/GSSG ratio detection kit to detect GSH/GSSG ratio; (G) SOD detection kit was used to detect SOD activity; (H) Gpx4 assay kit was used to analyse the activity of Gpx4. (I) Western blotting was used to detect the expression levels of ferroptosis‐related proteins, and the data are expressed as means ± SD, *n* = 4; Student's *t*‐test was used for comparison between the two groups, and one‐way analysis of variance was used for comparison between multiple groups; compared with the control group *p < 0.05, compared with the ox‐LDL group #*p* < 0.05.

MTT results showed that ox‐LDL caused damage to HUVECs, whereas the application of Ecd alleviated the damage to ox‐LDL‐treated HUVECs (Figure 2A). Our research showed that the Fe^2+^ content, MDA content, 4‐HNE level and ROS levels in ox‐LDL‐induced HUVECs were markedly increased, and the Fe^2+^, MDA, 4‐HNE and ROS levels in ox‐LDL‐induced HUVECs were significantly decreased after Ecd administration (Figure 2B–E). In contrast, after ox‐LDL induction, the GSH/GSSG ratio, SOD content, Gpx4 activity and the expression levels of ferroptosis‐related proteins (Gpx4, FTH1) in HUVECs prominently declined after ox‐LDL stimulation, and these effects were eliminated by Ecd (Figure 2F–I). In addition, ox‐LDL treatment increased the protein expression level of ACSL4 in HUVECs, whereas Ecd reversed the promotion of ox‐LDL on the protein expression level of ACSL4 (Figure 2I). These data indicate that Ecd can eliminate ferroptosis in ox‐LDL‐treated HUVECs.

In order to further explore the effect of Ecd on ferroptosis of HUVECs, HUVECs were treated with the ferroptosis activator erastin to induce ferroptosis, and then treated with Ecd. The results of the MTT assay showed that erastin inhibited the viability of HUVECs, whereas Ecd treatment reversed the inhibitory effect of erastin on the viability of HUVECs (Figure S1A). It is worth noting that the Fe^2+^ level, MDA content and ROS level were significantly higher in erastin‐treated HUVECs, whereas this effect was eliminated by Ecd treatment (Figure S1B–D). In contrast, erastin treatment inhibited Gpx4 enzyme activity in HUVECs, whereas Ecd treatment attenuated this effect (Figure S1E). Interestingly, mitochondrial morphology was changed in erastin‐treated HUVECs, such as mitochondrial swelling and mitochondrial cristae reduction or disappearance, whereas Ecd treatment restored mitochondrial morphology to normal (Figure S1F).

### 3.2 Ecd Inhibits Proinflammatory Cytokines Production in Ox‐LDL‐Treated HUVECs

**FIGURE 3:**
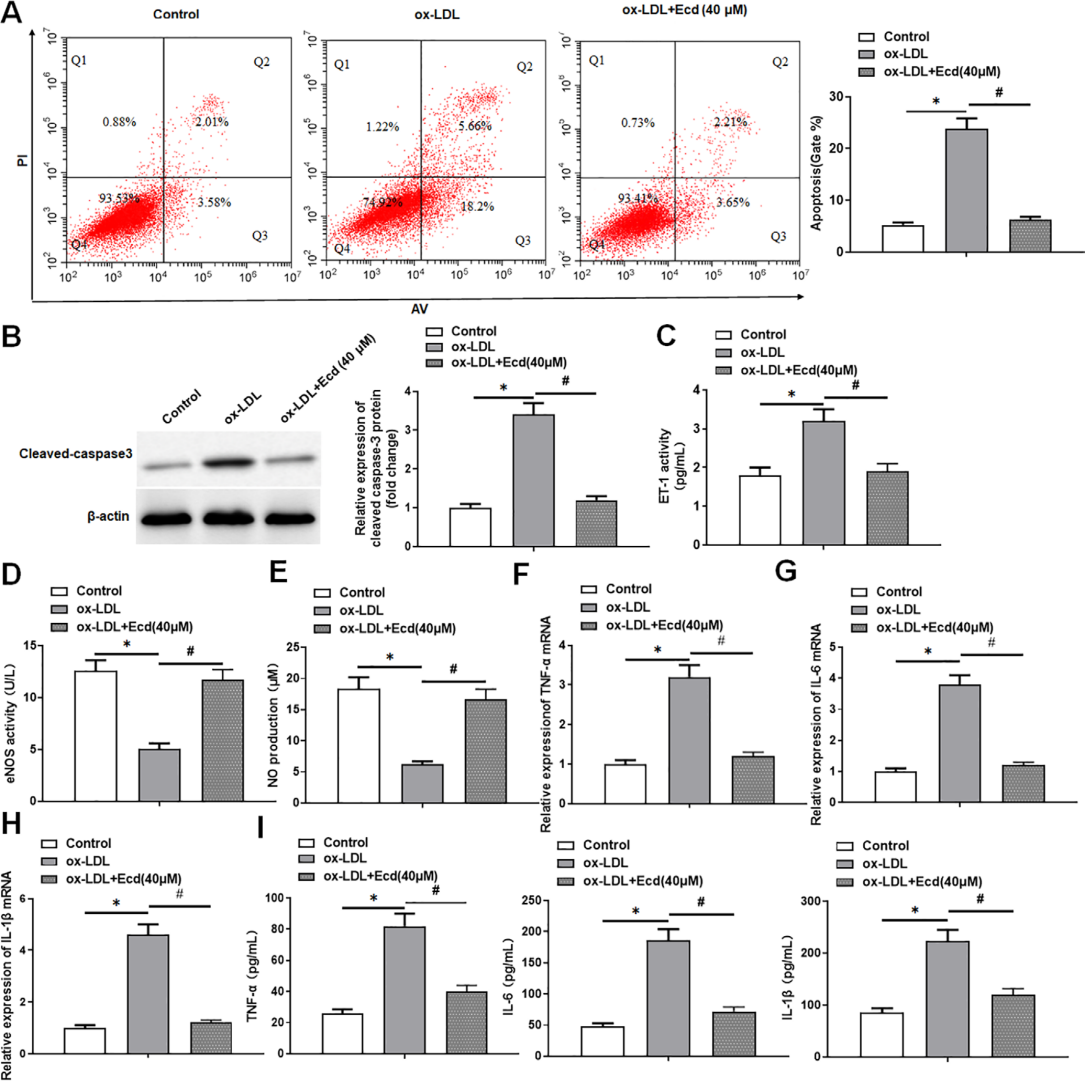
Ecd inhibited the production of proinflammatory cytokines in HUVECs. HUVECs were treated with Ecd (40 μM) for 24 h and then with 100 μM ox‐LDL for 24 h. (A) Flow cytometry was used to detect the apoptosis level of HUVECs induced by ox‐LDL; (B) Western blotting was used to detect the expression level of apoptosis‐related proteins; (C) ELISA was used to detect the level of endothelial cell injury marker ET‐1 in HUVECs supernatant; (D) The level of the endothelial cell injury marker eNOS was detected by ELISA in HUVECs supernatant. (E) The level of endothelial cell injury marker NO was detected by NO assay kit in HUVECs supernatant; (F) QPCR was used to detect the expression of inflammatory factor TNF‐α mRNA in HUVECs; (G) QPCR was used to detect the expression level of inflammatory factor IL‐6 mRNA in HUVECs; (H) QPCR was used to detect the expression level of inflammatory factor IL‐1β mRNA in HUVECs; (I) ELISA was used to detect the secretion of inflammatory factors in the supernatant of HUVECs; the data is expressed in means ± SD, *n* = 4; Student's *t* test was used for comparison between the two groups, and one‐way analysis of variance was used for comparison between multiple groups; compared with control group *p < 0.05, compared with ox‐LDL group #*p* < 0.05.

The results showed that ox‐LDL stimulation significantly promoted the apoptosis level of HUVECs and the expression of apoptosis‐related protein cleaved‐caspase3, while the use of Ecd improved the increased apoptosis level of HUVECs induced by ox‐LDL stimulation (Figure 3A,B). In addition, we observed that the levels of endothelial cell injury markers eNOS and NO were significantly decreased in HUVECs treated with ox‐LDL, and the level of ET‐1 was increased, whereas Ecd treatment attenuated the effect of ox‐LDL (Figure 3C–E). The results of qPCR and ELISA showed that the mRNA expression levels of inflammatory factors TNF‐α, IL‐6 and IL‐1β were significantly increased after HUVECs were stimulated by ox‐LDL. The secretion of TNF‐α, IL‐6 and IL‐1β in the cell supernatant was also significantly increased, while the application of Ecd reduced the production of inflammatory factors in ox‐LDL‐treated HUVECs (Figure 3F–I). These data suggest that Ecd may improve HUVECs injury by reducing ox‐LDL treatment‐induced HUVECs apoptosis and the inflammatory response.

### 3.3 Ecd Inhibits Ox‐LDL‐Treated Ferroptosis in HUVECs by Downregulating NCF2

**FIGURE 4:**
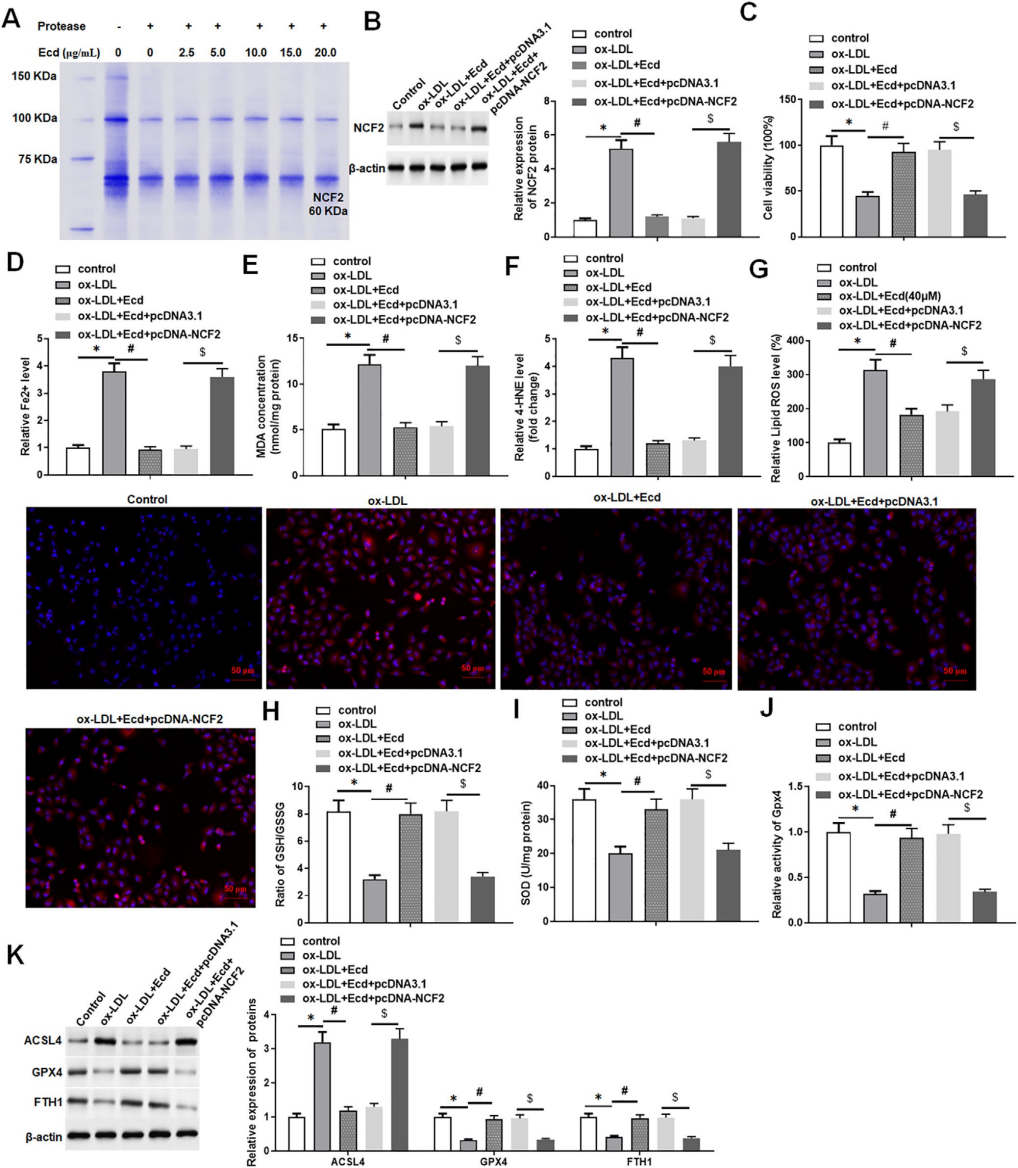
Ecd inhibits ox‐LDL‐induced ferroptosis in HUVECs by inhibiting NCF2. HUVECs were treated with 100 μM ox‐LDL for 24 h and Ecd (40 μM) for 24 h; Subsequently, HUVECs were transfected with 1.5 μg/mL pcDNA‐NCF2 expression vector, or negative control Vector (1.5 μg/mL); the transfected cells were cultured for 48 h for subsequent experiments. (A) DARTS verified the binding of Ecd and NCF2; (B) Western blotting was used to detect the expression of NCF2 protein; (C) MTT assay was used to detect the viability of HUVECs; (D) Iron assay kit to detect ferrous ion levels; (E) MDA detection kit to detect MDA content; (F) 4‐HNE detection kit to evaluate the level of 4‐HNE; (G) DCFH‐DA fluorescence probe method was used to detect lipid ROS levels (original magnification, 200×) Scale bars = 1 cm; (H) GSH/GSSG ratio detection kit was used to detect GSH/GSSG ratio; (I) SOD detection kit to detect SOD activity; (J) Gpx4 detection kit to analyse the activity of Gpx4; (K) Western blotting was used to detect the activity of ferroptosis‐related proteins; the data is expressed in means ± SD, *n* = 4; Student's *t* test was used for comparison between the two groups, and one‐way analysis of variance was used for comparison between multiple groups; compared with control group *p < 0.05, compared with ox‐LDL group #*p* < 0.05, compared with ox‐LDL + Ecd + pcDNA3.1 group $*p* < 0.05.

To explore the relationship between Ecd and NCF2, we used DARTS experiments to verify the binding of Ecd to NCF2. The results showed that the NCF2 band became weaker in the cell lysate treated with protease, and Ecd prevented the digestion of NCF2 by protease in a dose‐dependent manner (Figure 4A). Then, we detected the expression of NCF2 in HUVECs treated with ox‐LDL using Western blotting. The results showed that ox‐LDL treatment promoted NCF2 protein expression in HUVECs, whereas Ecd treatment inhibited the expression of NCF2 protein, indicating that NCF2 is a downstream effector molecule of Ecd (Figure 4B). To verify the effect of Ecd and NCF2 on the mechanism of AS, we explored whether the therapeutic effect of Ecd on ox‐LDL‐treated HUVECs injury can be changed by overexpression of NCF2. The results showed that Ecd attenuated the inhibition of ox‐LDL‐treated HUVECs viability, while overexpression of NCF2 reversed this effect (Figure 4C). After overexpression of NCF2, the weakened effect of Ecd on ox‐LDL‐induced ferroptosis in HUVECs was restored, as evidenced by the increase in Fe^2+^ content, MDA content, 4‐HNE level and ROS level, as well as a decrease in the GSH/GSSG ratio, SOD content, Gpx4 activity and the expression levels of ferroptosis‐related proteins (Gpx4 and FTH1) (Figure 4D–K). These results suggest that overexpression of NCF2 attenuates the inhibition of ferroptosis by Ecd in ox‐LDL‐treated HUVECs.

### 3.4 Ecd Inhibits Ox‐LDL‐Treated HUVECs Damage by Upregulating NCF2

**FIGURE 5:**
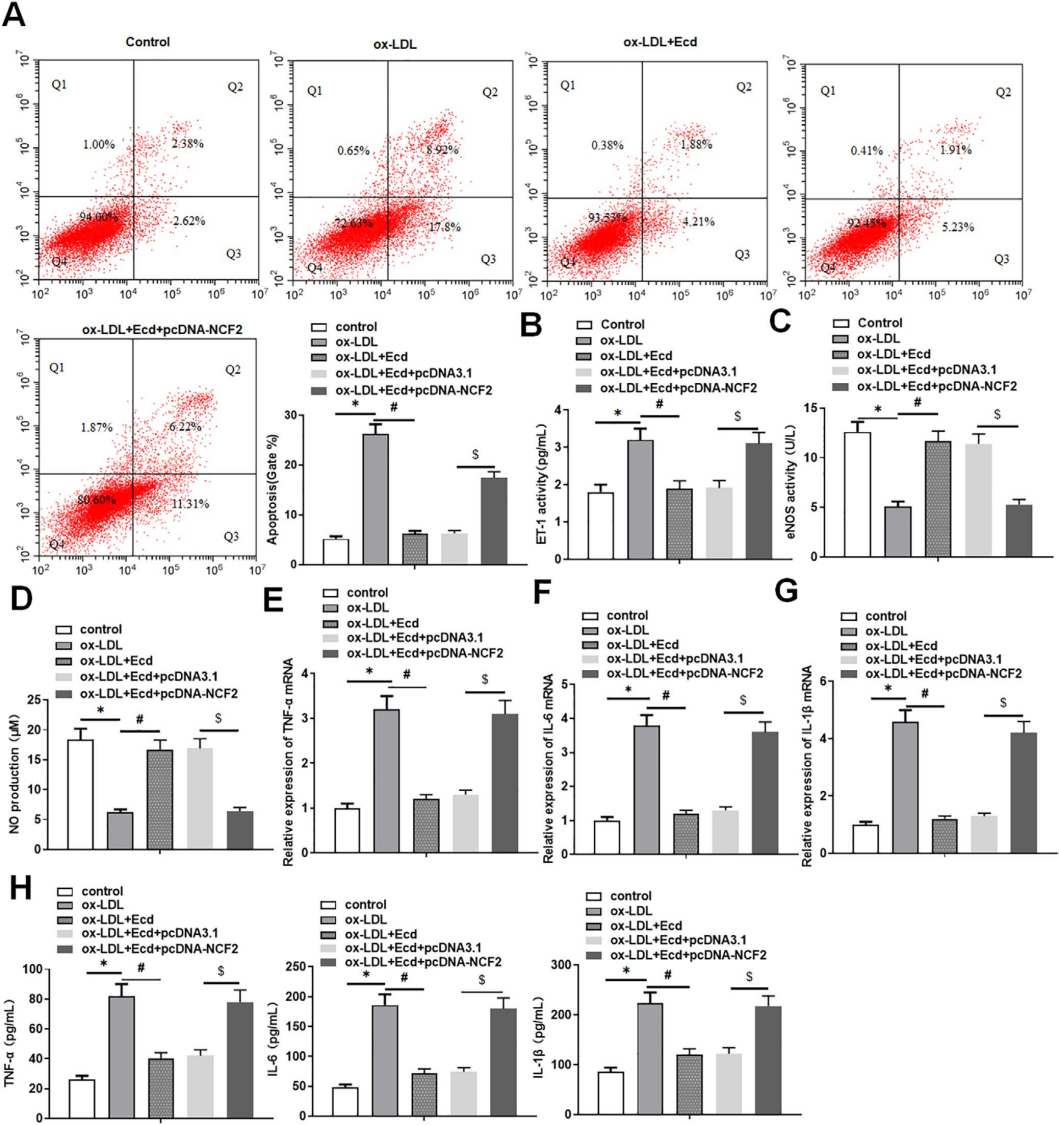
Ecd reduces HUVECs damage by inhibiting NCF2. HUVECs were treated with 100 μM ox‐LDL for 24 h or Ecd (40 μM) for 24 h. Subsequently, HUVECs were transfected with 1.5 μg/mL pcDNA‐NCF2 expression vector or a negative control vector (1.5 μg/mL). The transfected cells were cultured for 48 h for subsequent experiments. (A) Flow cytometry was used to detect the apoptosis level of HUVECs. (B) ELISA was used to detect the level of endothelial cell injury marker ET‐1 in HUVECs supernatant. (C) The level of the endothelial cell injury marker eNOS was detected by ELISA in HUVECs supernatant. (D) The level of endothelial cell injury marker NO was detected by NO assay kit in HUVECs supernatant; (E) QPCR was used to detect the expression of inflammatory factor TNF‐α mRNA in HUVECs; (F) QPCR was used to detect the expression level of inflammatory factor IL‐6 mRNA in HUVECs; (G) QPCR was used to detect the expression level of inflammatory factor IL‐1β mRNA in HUVECs; (H) ELISA was used to detect the secretion of inflammatory factors in the supernatant of HUVECs; the data is expressed in means ± SD, *n* = 4; Student's *t* test was used for comparison between the two groups, and one‐way analysis of variance was used for comparison between multiple groups; compared with control group *p < 0.05, compared with ox‐LDL group #*p* < 0.05, compared with ox‐LDL + Ecd + pcDNA3.1 group $*p* < 0.05.

Flow cytometry was used to analyse the levels of apoptosis in HUVECs under different treatments. We found that Ecd attenuated the apoptosis of ox‐LDL‐treated HUVECs, while treatment with pcDNA‐NCF2 reversed this effect (Figure 5A). In addition, we found that the effect of ox‐LDL stimulation on the decrease in eNOS and NO levels and the increase in ET‐1 was attenuated by Ecd, while overexpression of NCF2 restored the effect of Ecd on eNOS, NO and ET‐1 levels in ox‐LDL‐treated HUVECs (Figure 5B–D). The results of qPCR and ELISA showed that the mRNA expression levels of inflammatory factors TNF‐α, IL‐6 and IL‐1β and the secretion of TNF‐α, IL‐6 and IL‐1β in the cell supernatant were reduced after the application of Ecd in ox‐LDL‐treated HUVECs, whereas the inhibitory effects of Ecd on ox‐LDL‐treated inflammatory factors in HUVECs were effectively reversed by transfection with pcDNA‐NCF2 (Figure 5E–H). These results indicated that overexpression of NCF2 could reverse the inhibitory effects of Ecd on ox‐LDL‐induced apoptosis and inflammation in HUVECs.

### 3.5 NCF2 Promotes Ferroptosis in HUVECs by Inhibiting the PI3K/Akt/Nrf2 Pathway

**FIGURE 6:**
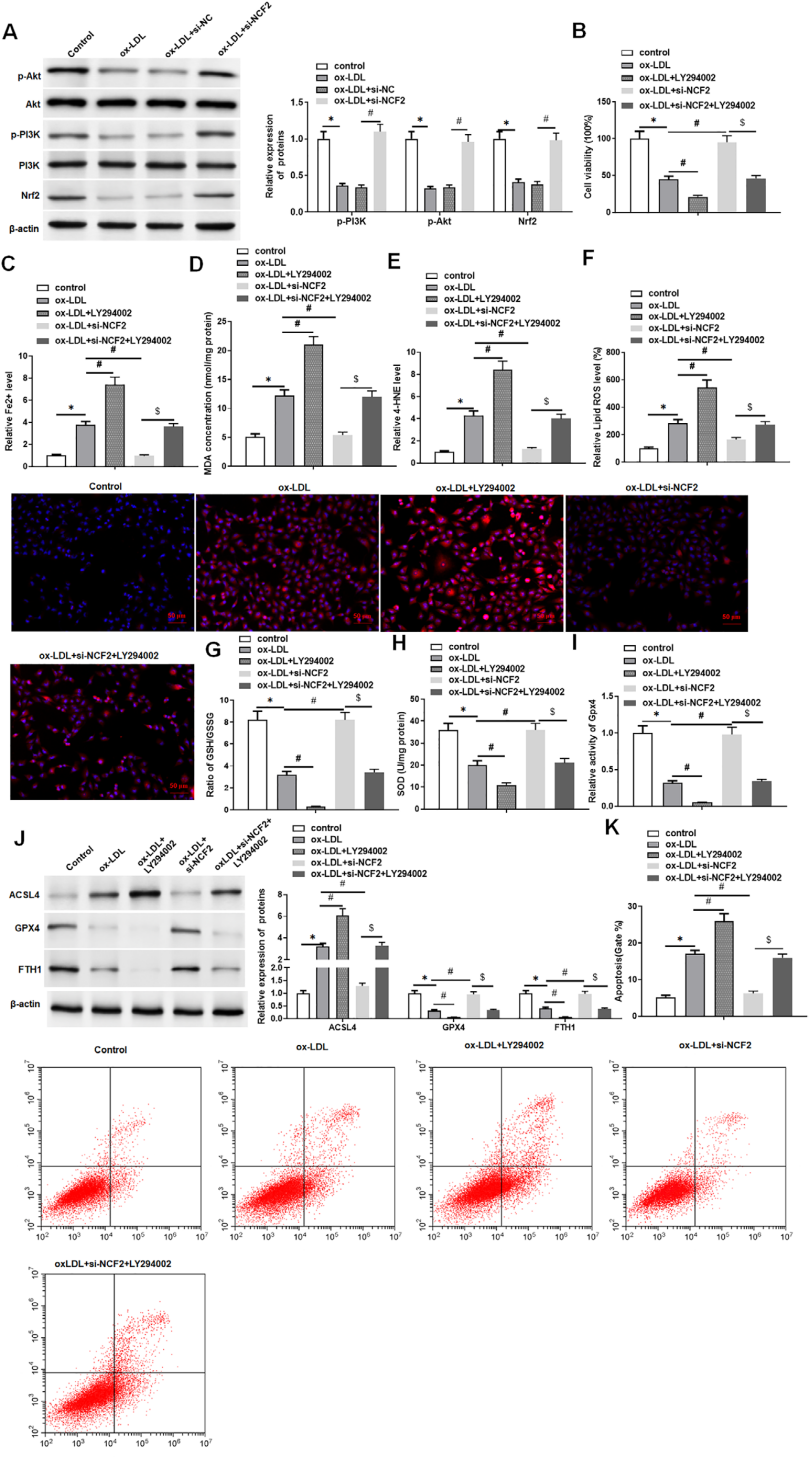
NCF2 promotes ox‐LDL‐treated ferroptosis in HUVECs by inhibiting PI3K/Akt/Nrf2 pathway. HUVECs were treated with 100 μM ox‐LDL for 24 h, followed by treatment with 3 μM LY294002 for 24 h, transfection with 25 nM si‐NCF2 or transfection with 25 nM si‐NCF2 and treatment with 3 μM LY294002 for 24 h. (A) Western blotting was used to detect the expression levels of the PI3K/Akt/Nrf2 pathway‐related proteins; (B) MTT assay was used to detect HUVECs viability; (C) Iron assay kit to detect ferrous ion level; (D) MDA detection kit to detect MDA content; (E) 4‐HNE detection kit was used to evaluate the level of 4‐HNE; (F) DCFH‐DA fluorescent probe method was used to detect lipid ROS levels (original magnification, 200×) Scale bars = 1 cm; (G) GSH/GSSG ratio detection kit was used to detect GSH/GSSG ratio; (H) SOD detection kit to detect SOD activity; (I) Gpx4 detection kit to analyse the activity of Gpx4; (J) Western blotting was used to detect the activity of ferroptosis‐related proteins; (K) Flow cytometry was used to detect the apoptosis level of HUVECs; the data is expressed in means ± SD, *n* = 4; Student's *t* test was used for comparison between the two groups, and one‐way analysis of variance was used for comparison between multiple groups; compared with control group *p < 0.05, compared with ox‐LDL group #*p* < 0.05, compared with ox‐LDL + si‐NCF2 group $*p* < 0.05.

To explore the regulatory effect of NCF2 on ferroptosis in ox‐LDL‐treated HUVECs, we studied the changes in the PI3K/Akt/Nrf2 signalling pathway in HUVECs based on NCF2. We found that after ox‐LDL stimulation of HUVECs, the expression levels of p‐PI3K, p‐Akt and Nrf2 were significantly decreased, while after transfection of si‐NCF2, the inhibitory effect of ox‐LDL stimulation on the expression levels of p‐PI3K, p‐Akt and Nrf2 was restored (Figure 6A). MTT results showed that the application of the PI3K/Akt/Nrf2 inhibitor LY294002 reduced the viability of ox‐LDL‐treated HUVECs, and the transfection of si‐NCF2 increased the viability of ox‐LDL‐treated HUVECs, while LY294002 reversed the effect of si‐NCF2 on the viability of ox‐LDL‐treated HUVECs (Figure 6B). In addition, LY294002 further increased ferroptosis in ox‐LDL‐treated HUVECs, including increased Fe^2+^ content, MDA content, 4‐HNE level, ROS level and ACSL4 expression, as well as decreased GSH/GSSG ratio, SOD content, Gpx4 activity and the levels of ferroptosis‐related proteins (Gpx4 and FTH1). si‐NCF2 reduced ferroptosis in ox‐LDL‐treated HUVECs, while LY294002 reversed the inhibitory effect of si‐NCF2 on ferroptosis in ox‐LDL‐treated HUVECs (Figure 6C–J). The results of flow cytometry analysis showed that si‐NCF2 reduced the apoptosis of HUVECs, while the application of LY294002 weakened the inhibition of apoptosis by si‐NCF2 in ox‐LDL‐treated HUVECs (Figure 6K). These results indicate that NCF2 could promote ferroptosis in ox‐LDL‐treated HUVECs by inhibiting the PI3K/Akt/Nrf2 pathway.

### 3.6 Ecd Inhibits Ferroptosis of Ox‐LDL‐Treated HUVECs by Inhibiting NCF2 to Activate the PI3K/Akt/Nrf2 Pathway

**FIGURE 7:**
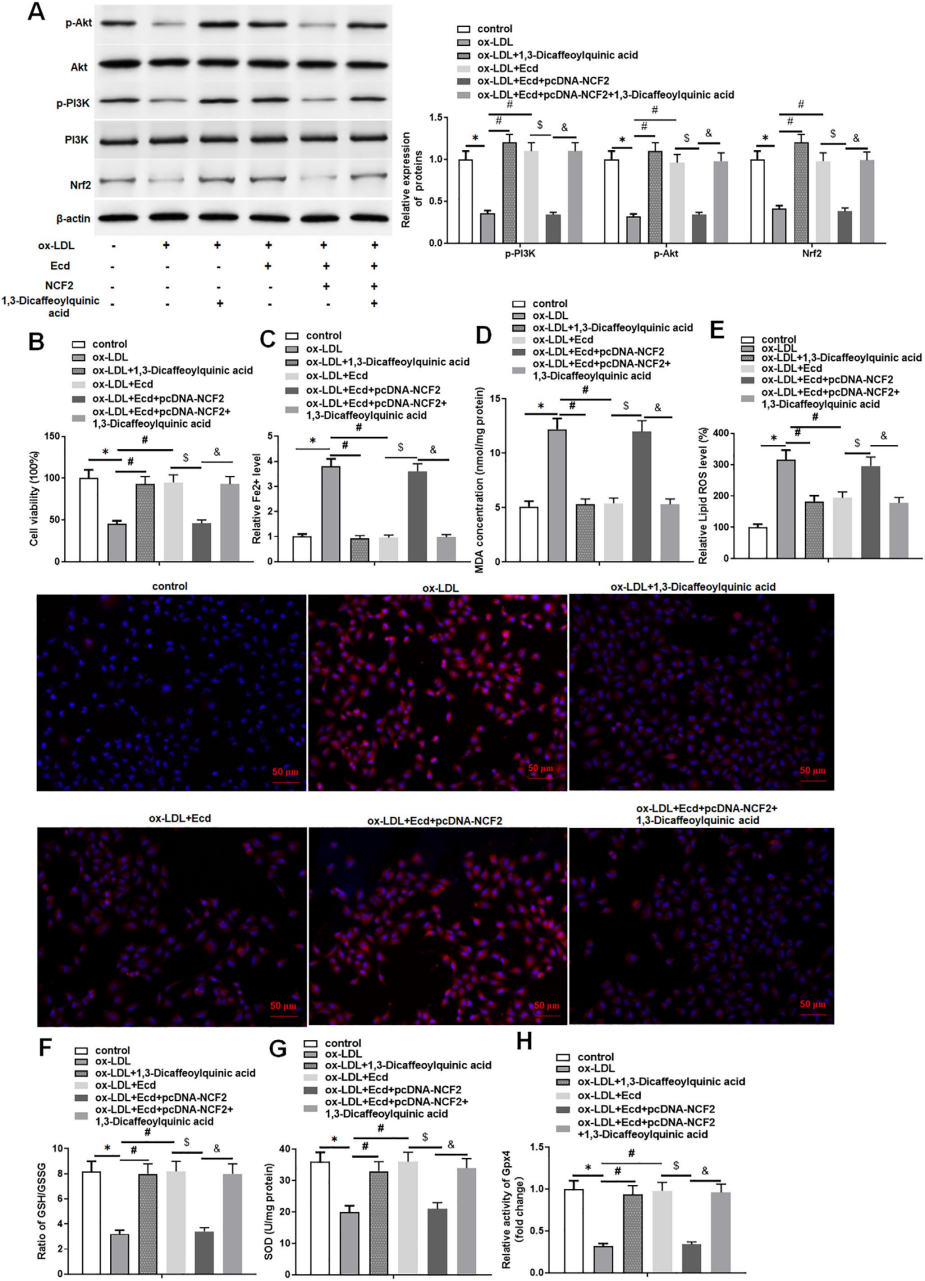
Ecd inhibits ferroptosis of ox‐LDL‐treated HUVECs by inhibiting NCF2 to activate the PI3K/Akt/Nrf2 pathway. HUVECs were treated with 100 μM ox‐LDL for 24 h, followed by treatment with 10 mM 1,3‐Dicaffeoylquinic acid for 24 h, Ecd (40 μM) for 24 h, Ecd treatment and transfection of 1.5 μg/mL pcDNA‐NCF2 vector, Ecd treatment and transfection of 1.5 μg/mL pcDNA‐NCF2 vector, and treatment with 10 mM 1,3‐Dicaffeoylquinic acid for 24 h. (A) Western blotting was used to detect the expression levels of PI3K/Akt/Nrf2 pathway‐related proteins; (B) MTT assay was used to detect HUVECs viability; (C) Iron assay kit to detect ferrous ion levels; (D) MDA detection kit to detect MDA content; (E) DCFH‐DA fluorescent probe method was used to detect lipid ROS levels (original magnification, 200×) Scale bars = 1 cm; (F) GSH/GSSG ratio detection kit to detect GSH/GSSG ratio; (G) SOD detection kit was used to detect SOD activity; (H) Gpx4 detection kit was used to analyse the activity of Gpx4; the data is expressed in means ± SD, *n* = 4; Student's *t* test was used for comparison between the two groups, and one‐way analysis of variance was used for comparison between multiple groups; compared with control group *p < 0.05, compared with ox‐LDL group #*p* < 0.05, compared with ox‐LDL + Ecd group $*p* < 0.05; compared with ox‐LDL + Ecd + pcDNA‐NCF2 group &*p* < 0.05.

We used 1,3‐Dicaffeoylquinic acid, a specific activator of the PI3K/Akt/Nrf2 pathway, to further investigate the molecular mechanism by which this pathway is involved in regulating AS. As shown in Figure 7A, both Ecd treatment and 1,3‐Dicaffeoylquinic acid treatment significantly increased the expression levels of p‐PI3K, p‐Akt and Nrf2 in ox‐LDL‐treated HUVECs. Transfection with pcDNA‐NCF2 attenuated the effect of Ecd on the expression of p‐PI3K, p‐Akt and Nrf2 in ox‐LDL‐treated HUVECs. In addition, 1,3‐Dicaffeoylquinic acid reversed the effects of Ecd and pcDNA‐NCF2 simultaneously on the expression levels of p‐PI3K, p‐Akt and Nrf2 in ox‐LDL‐treated HUVECs. The MTT results showed that 1,3‐Dicaffeoylquinic acid treatment promoted the viability of ox‐LDL‐treated HUVECs, which was consistent with the results of Ecd treatment. Overexpression of NCF2 reversed the effect of Ecd on the viability of ox‐LDL‐treated HUVECs, whereas co‐application of 1,3‐Dicaffeoylquinic acid reversed the effect of Ecd and pcDNA‐NCF2 on the viability of ox‐LDL‐treated HUVECs (Figure 7B). In addition, 1,3‐Dicaffeoylquinic acid and Ecd had the same effect on ferroptosis induced by ox‐LDL in HUVECs, that is, inhibiting ferroptosis. Overexpression of NCF2 rescued the inhibitory effect of Ecd on ferroptosis in ox‐LDL‐treated HUVECs, while 1,3‐Dicaffeoylquinic acid reversed the combined effect of Ecd and NCF2 on ferroptosis in ox‐LDL‐treated HUVECs, the decrease in Fe^2+^ content, MDA concentration, and ROS level, as well as the increase in the GSH/GSSG ratio, SOD concentration and Gpx4 activity (Figure 7C–H). In summary, these results confirm that Ecd inhibits NCF2 and ox‐LDL‐induced ferroptosis in HUVECs by activating the PI3K/Akt/Nrf2 pathway.

### 3.7 Ecd Activates the PI3K/Akt/Nrf2 Pathway by Inhibiting NCF2, Thereby Reducing Damage to HUVEC Cells

**FIGURE 8:**
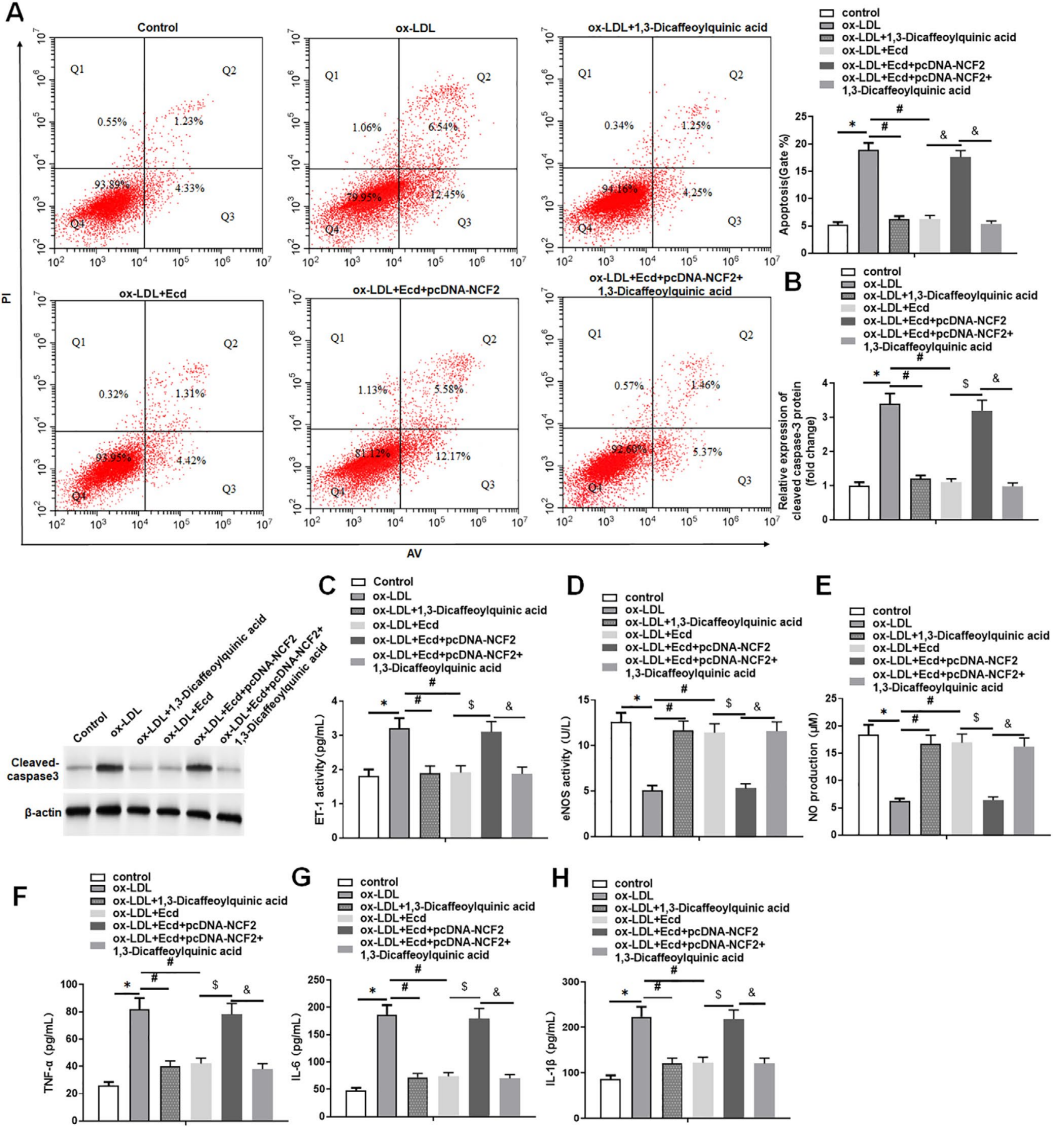
Ecd activates the PI3K/Akt/Nrf2 pathway by inhibiting NCF2, thereby reducing HUVECs damage. HUVECs were treated with 100 μM ox‐LDL for 24 h, followed by treatment with 10 mM 1,3‐Dicaffeoylquinic acid for 24 h, Ecd (40 μM) for 24 h, Ecd treatment and transfection of 1.5 μg/mL pcDNA‐NCF2 vector, and treatment with 10 mM 1,3‐Dicaffeoylquinic acid for 24 h. (A) Flow cytometry was used to detect the apoptosis level of HUVECs; (B) Western blotting was used to detect the expression level of apoptosis‐related proteins; (C) ELISA was used to detect the level of endothelial cell injury marker ET‐1 in HUVECs supernatant; (D) The level of the endothelial cell injury marker eNOS was detected by ELISA in HUVECs supernatant of. (E) The level of the endothelial cell injury marker NO was detected using an NO assay kit in HUVECs supernatant. (F–H) The secretion levels of inflammatory factors TNF‐α, IL‐6 and IL‐1βwere detected by ELISA in HUVECs supernatant, and the data are expressed as means ± SD, *n* = 4. Student's *t*‐test was used for comparison between the two groups, and one‐way analysis of variance was used for comparison between multiple groups; compared with the control group *p < 0.05, compared with ox‐LDL + Ecd group #*p* < 0.05; compared with ox‐LDL + Ecd group, $*p* < 0.05; compared with ox‐LDL + Ecd + pcDNA‐NCF2 group &*p* < 0.05.

The apoptosis rate of HUVECs after different treatments was analysed using flow cytometry. We found that 1,3‐Dicaffeoylquinic acid and Ecd inhibited apoptosis in ox‐LDL‐treated HUVECs, while treatment with pcDNA‐NCF2 reversed the inhibitory effect of Ecd on apoptosis. The application of 1,3‐Dicaffeoylquinic acid under the combined action of Ecd and NCF2 reversed the effect of Ecd and NCF2 on the apoptosis of ox‐LDL‐treated HUVECs (Figure 8A). Furthermore, the results showed that both 1,3‐dicaffeoylquinic acid and Ecd increased eNOS and NO levels in ox‐LDL‐treated HUVECs and inhibited ET‐1 levels. Overexpression of NCF2 attenuated this effect of Ecd, whereas 1,3‐dicaffeoylquinic acid reversed the effects of simultaneous treatment of Ecd and overexpression of NCF2 on eNOS, NO and ET‐1 levels in ox‐LDL‐treated HUVECs (Figure 8B–D). 1,3‐Dicaffeoylquinic acid treatment and Ecd treatment significantly reduced the secretion of inflammatory cytokines induced by ox‐LDL treatment, including IL‐1, IL‐1β and TNF‐α, while pcDNA‐NCF2 treatment reversed the inhibitory effect of Ecd treatment on the secretion of inflammatory cytokines. The application of 1,3‐Dicaffeoylquinic acid under the co‐treatment of Ecd and NCF2 reversed the effect of the interaction between Ecd and NCF2 on the secretion of inflammatory cytokines (Figure 8E–H). In summary, these results indicate that Ecd inhibition of NCF2 attenuates ox‐LDL stimulation of HUVECs injury by activating the PI3K/Akt/Nrf2 pathway.

### 3.8 Ecd Treatment Inhibits Ferroptosis in AS Mice by Downregulating NCF2

Firstly, two groups of C57BL/6 mice were intravenously injected with saline and Ecd (50 mg/kg) every 4 days for half a month to evaluate the in vivo safety of Ecd. The results showed that the body weight changes of Ecd‐treated mice were similar to those of saline‐treated mice (Figure S3A). Ecd treatment also did not affect blood oxygen partial pressure and oxygen saturation in mice (Figure S3B). Additionally, the levels of liver function biomarkers (alanine aminotransferase (ALT) and aspartate aminotransferase [AST]) and renal function biomarkers (blood urea nitrogen [BUN] and creatinine [Cr]), as well as inflammatory cytokines (TNF‐α, IL‐6 and IL‐1β) in the serum of mice in the Ecd treatment group were comparable to those in the saline treatment group (Figure S3C–E). Furthermore, the blood glucose level of Ecd‐treated mice was the same as that of the control group, and the weights of the liver, kidney and spleen of Ecd‐treated mice were similar to those of the control group (Figure S3F,G).

**FIGURE 9:**
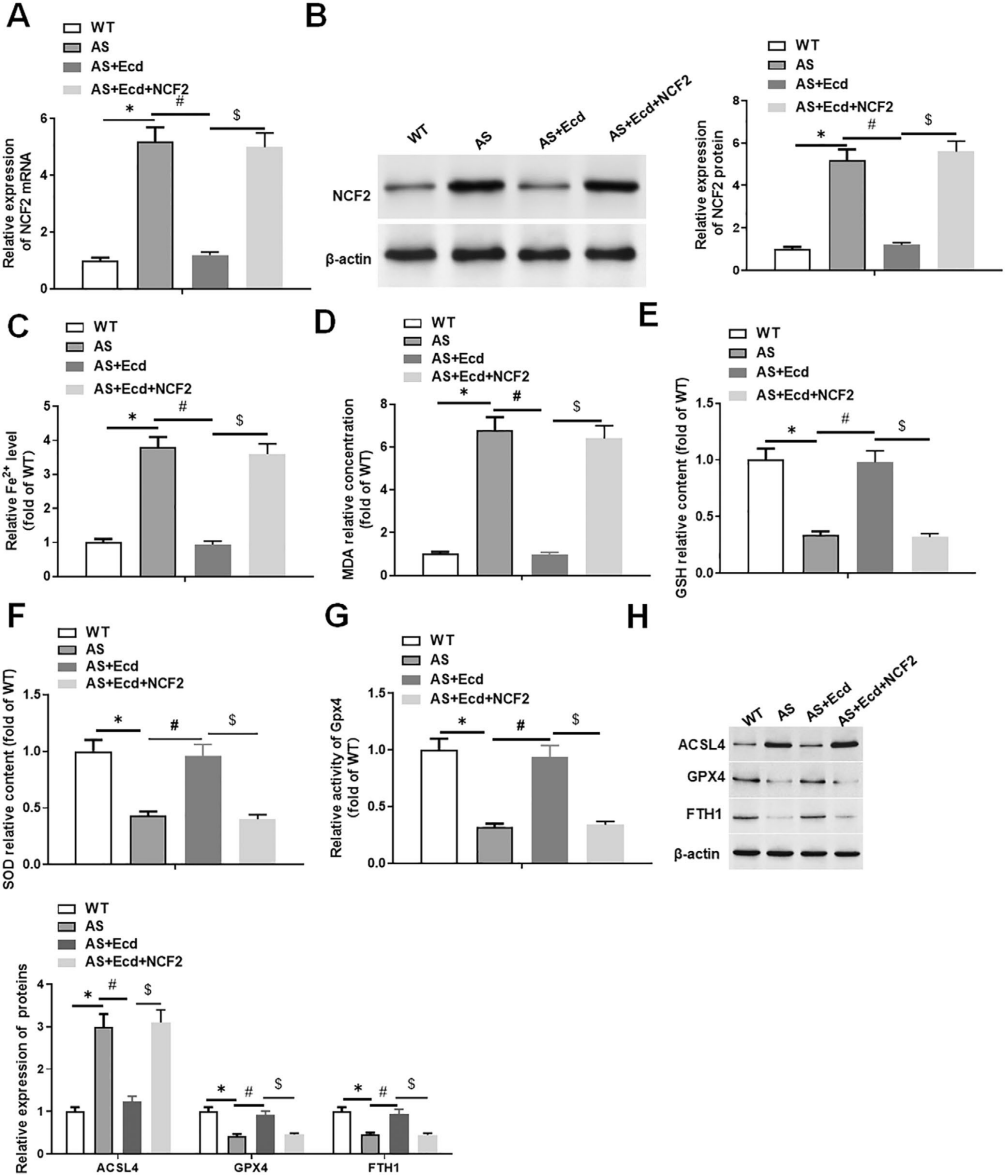
Ecd treatment inhibits ferroptosis in AS mice by downregulating NCF2. ApoE^−/−^ mice were fed a high‐fat diet for 10 weeks to establish the AS mouse model. During this period, Ecd (25 mg/kg) was fed daily, and 500 μL of NCF2 lentivirus overexpression vector (2 μg/mL) was injected into the tail vein every 2 weeks. (A) QPCR was used to detect the expression of NCF2 mRNA in mouse arterial tissue; (B) Western blotting was used to detect the expression level of NCF2 protein in mouse arterial tissue; (C) Iron determination kit was used to detect the level of ferrous ions in mouse tissue homogenate; (D) MDA detection kit was used to detect MDA content in mouse tissue homogenate; (E) The GSH level in mouse tissue homogenate was detected using GSH detection kit (F) SOD detection kit was used to detect SOD activity in mouse tissue homogenate; (G) Gpx4 detection kit was used to analyse the activity of Gpx4 in mouse tissue homogenate; (H) Western blotting was used to detect the expression level of ferroptosis key protein in mouse arterial tissue, and the data are expressed as means ± SD, *n* = 8; Student's *t*‐test was used for comparison between the two groups, and one‐way analysis of variance was used for comparison between multiple groups; compared with WT group *p < 0.05, compared with AS group #*p* < 0.05, compared with AS+Ecd group $*p* < 0.05.

ApoE^−/−^ mice were fed a high‐fat diet for 10 weeks to establish the AS mouse model. During this period, Ecd (25 mg/kg) was administered daily, or 500 μL of the NCF2 lentivirus overexpression vector (2 μg/mL) was injected into the tail vein at the same time. We found that NCF2 was upregulated in the tissues of AS mice, and Ecd treatment inhibited the expression of NCF2, whereas NCF2 reversed the inhibitory effect of Ecd on NCF2 expression (Figure 9A,B). The levels of arterial ferroptosis in AS mice were increased, which was manifested by the increase in Fe^2+^ concentration and MDA concentration, as well as the decrease in GSH concentration, SOD concentration and Gpx4 activity, while treatment with Ecd inhibited arterial ferroptosis in AS mice. In addition, NCF2 reversed the inhibitory effect of Ecd treatment on arterial ferroptosis in mice (Figure 9C–G). Western blotting results showed that the expression of the ferroptosis key proteins GPX4 and FTH1 was significantly decreased in the arterial tissue of AS mice, and ACSL4 was decreased. Ecd treatment promoted the expression of GPX4 and FTH1 and inhibited ACSL4, whereas overexpression of NCF2 reversed the effect of Ecd (Figure 9H). Thus, Ecd treatment inhibits ferroptosis in mice by downregulating NCF2.

### 3.9 Ecd Treatment Attenuates AS Lesions in ApoE ^−/−^ Mice by Downregulating NCF2

**FIGURE 10:**
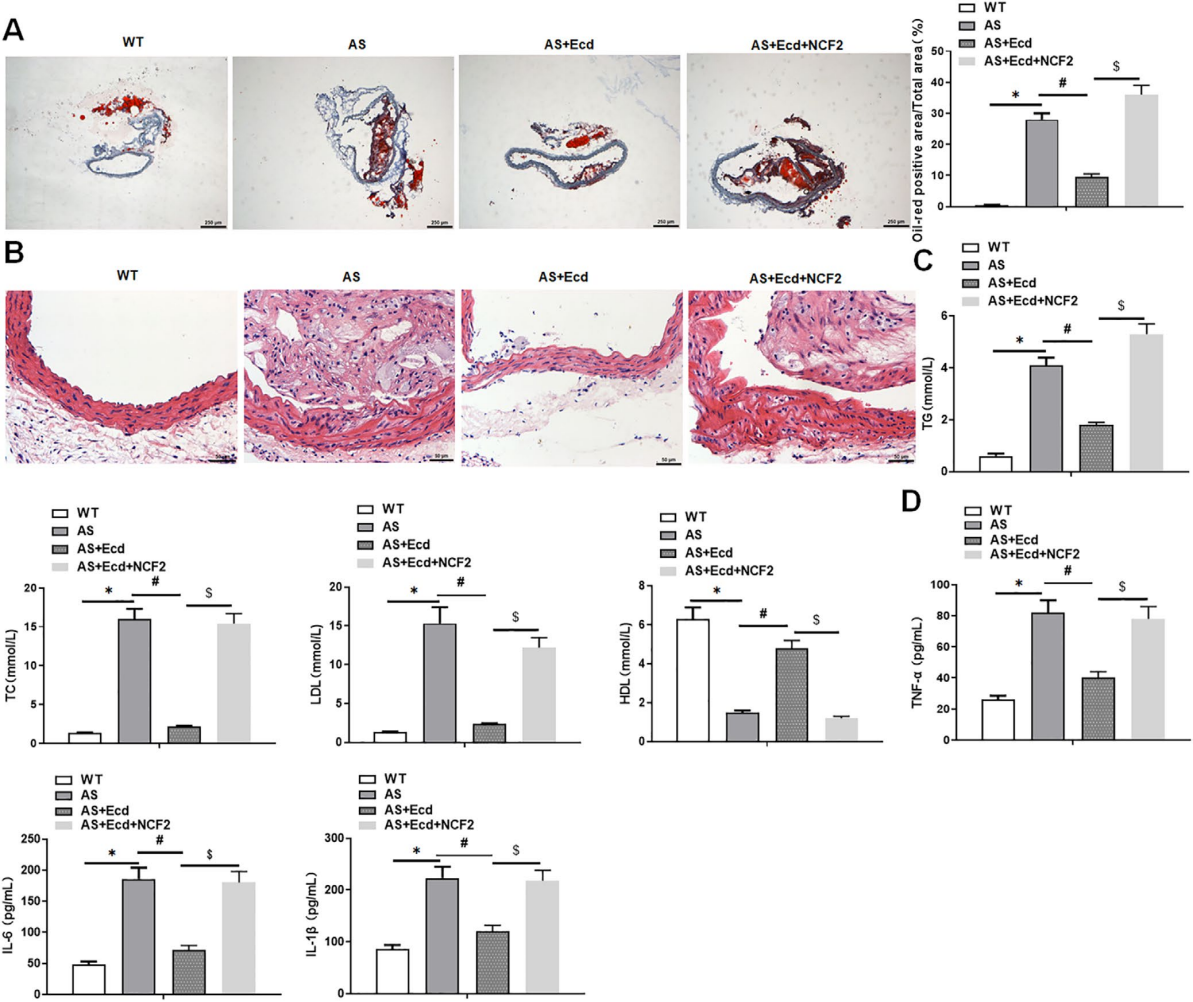
Ecd attenuates HFD‐induced AS in ApoE^−/−^ mice by inhibiting NCF2. ApoE^−/−^ mice were fed a high‐fat diet for 10 weeks to establish the AS mouse model. During this period, Ecd (25 mg/kg) was fed daily, and 500 μL of NCF2 lentivirus overexpression vector (2 μg/mL) was injected into the tail vein every 2 weeks. (A) Oil red O staining was used to detect the degree of arterial plaque (original magnification, 40×) Scale bars = 10 cm; (B) HE staining for pathological analysis of arterial tissue (original magnification, 200×) Scale bars = 1 cm; (C) Detection of serum TC, TG, LDL and HDL levels; (D) The secretion levels of inflammatory factors TNF‐α, IL‐6 and IL‐1β were detected by ELISA, and the data are expressed as means ± SD, *n* = 8. Student's *t*‐test was used for comparison between the two groups, and one‐way analysis of variance was used for comparison between multiple groups; compared with WT group *p < 0.05, compared with AS group #*p* < 0.05, compared with AS+Ecd group $*p* < 0.05.

Next, we explored the effect of Ecd on AS progression. Oil red O staining of aortic tissue showed that the lesion area of AS mice was larger than that of WT mice. In addition, similar to the ATO treatment group, Ecd treatment significantly reduced the aortic lesion area of AS mice, whereas NCF2 reversed the inhibitory effect of Ecd treatment on the lesion area of AS mice (Figure 10A and Figure S2A). The results of HE staining showed that the lesion area of the aortic intima in the AS group was larger and contained a larger lesion necrosis area, while Ecd administration reduced the lesion and necrotic areas, and overexpression of NCF2 reversed the effect of Ecd administration (Figure 10B). Subsequently, serum TG, TC, LDL and HDL levels were measured to determine the effects of Ecd on blood lipid levels. Compared to the WT group, the AS group had higher serum TG, TC and LDL levels and lower HDL levels, whereas Ecd and ATO treatment decreased TG, TC and LDL levels and increased HDL levels. These changes in Ecd treatment were reversed by overexpression of NCF2 (Figure 10C and Figure S2B). We then detected the concentrations of several inflammatory cytokines in the serum of AS mice. The results showed that the concentrations of IL‐6, IL‐1β and TNF‐α were significantly reduced in AS mice treated with ATO and Ecd, whereas overexpression of NCF2 restored the effect of Ecd (Figure 10D and Figure S2C). Taken together, these results suggest that Ecd attenuates AS and inflammation in ApoE^−/−^ mice.

## 4 Discussion

The results of this study demonstrate that Ecd could be used as a new drug for the treatment of AS. As the main component of a variety of Chinese herbal medicines, Ecd relieves hyperglycemia and hyperlipidemia, regulates immunity and protects HUVECs from apoptosis [21]. A study reported that Ecd activates autophagy in chondrocytes to alleviate osteoarthritis [37]. Ecd also protects osteoblasts from apoptosis [38]. Interestingly, under focal cerebral ischemia–reperfusion conditions, Ecd attenuates oxidative stress and increases rat survival [39]. Similarly, Ecd significantly reduced the levels of MDA and ROS in H_2_O_2_‐stimulated rat B35 neuroblastoma cells [40]. In this study, we found that Ecd inhibited ferroptosis of HUVECs induced by ox‐LDL, which was manifested by a decrease in MDA and ROS, as well as an increase in SOD and GSH. In addition, we found that compared with normal mice, AS mice had more severe aortic lesions, and Ecd treatment reduced the aortic pathological changes in AS model mice. Thus, Ecd has a protective effect against AS.

There is evidence that NCF2 can be used as a key gene for AS [41], which is also associated with a variety of diseases and acts as a negative factor in diseases including cancer, leukaemia [42], myocardial infarction [43], systemic lupus erythematosus [44] and so on. Several studies have found that NCF2 is involved in a variety of physiological and pathological processes such as inflammation regulation [45], cell death [46], oxidative stress [30] and ferroptosis [31]. After NCF2 interference, it inhibits the production of ROS, thereby exerting significant inhibitory hypertrophic activity and having a considerable clinical effect [47]. In addition, SRF promotes liver fibrosis by inducing the transcriptional activation of NCF1/NCF2 to promote ROS production [48]. In this study, we found that NCF2 was upregulated in ox‐LDL‐treated HUVECs and the arterial tissues of AS model mice. Interfering with NCF2 inhibited ferroptosis in ox‐LDL‐treated HUVECs and alleviated arterial ferroptosis and inflammatory responses in AS mice. In addition, Ecd inhibited the expression of NCF2.

The PI3K/Akt/Nrf2 signalling pathway is closely related to inflammation, oxidative stress, cell necrosis and apoptosis [49]. Several studies have reported that activation of this pathway can reduce organ damage and inflammatory responses, including kidney injury [50], brain injury [51], lung injury [52], colitis [53], etc. A previous study has found that Rehmannioside A exerts neuroprotective effects by inhibiting ferroptosis and activating the PI3K/Akt/Nrf2 signalling pathway, thereby improving cognitive dysfunction after cerebral ischemia [16]. Hesperetin upregulated HO‐1 levels by triggering the PI3K/AKT/Nrf2 pathway to alleviate OA‐induced ROS overproduction and hepatotoxicity [54]. Sipeimine improved PM2.5‐induced lung injury by activating the PI3K/Akt/Nrf2 pathway and inhibiting ferroptosis [55]. Similarly, the study also found that geraniol inhibited ox‐LDL‐treated inflammation and oxidative stress in HUVECs by upregulating the PI3K/Akt/Nrf2 pathway. The upregulation of PI3K can alleviate AS mouse oxidant‐induced endothelial cell damage and inhibit AS progression [56]. In this study, we found that Ecd inhibited ox‐LDL‐treated HUVECs ferroptosis and injury by upregulating the PI3K/Akt/Nrf2 pathway and reducing the secretion of inflammatory factors, thereby alleviating AS‐induced arterial injury. In addition, the effect of Ecd was reversed by NCF2 overexpression. Our results showed that the therapeutic mechanism of Ecd in AS is related to the PI3K/Akt/Nrf2 pathway.

**FIGURE 11:**
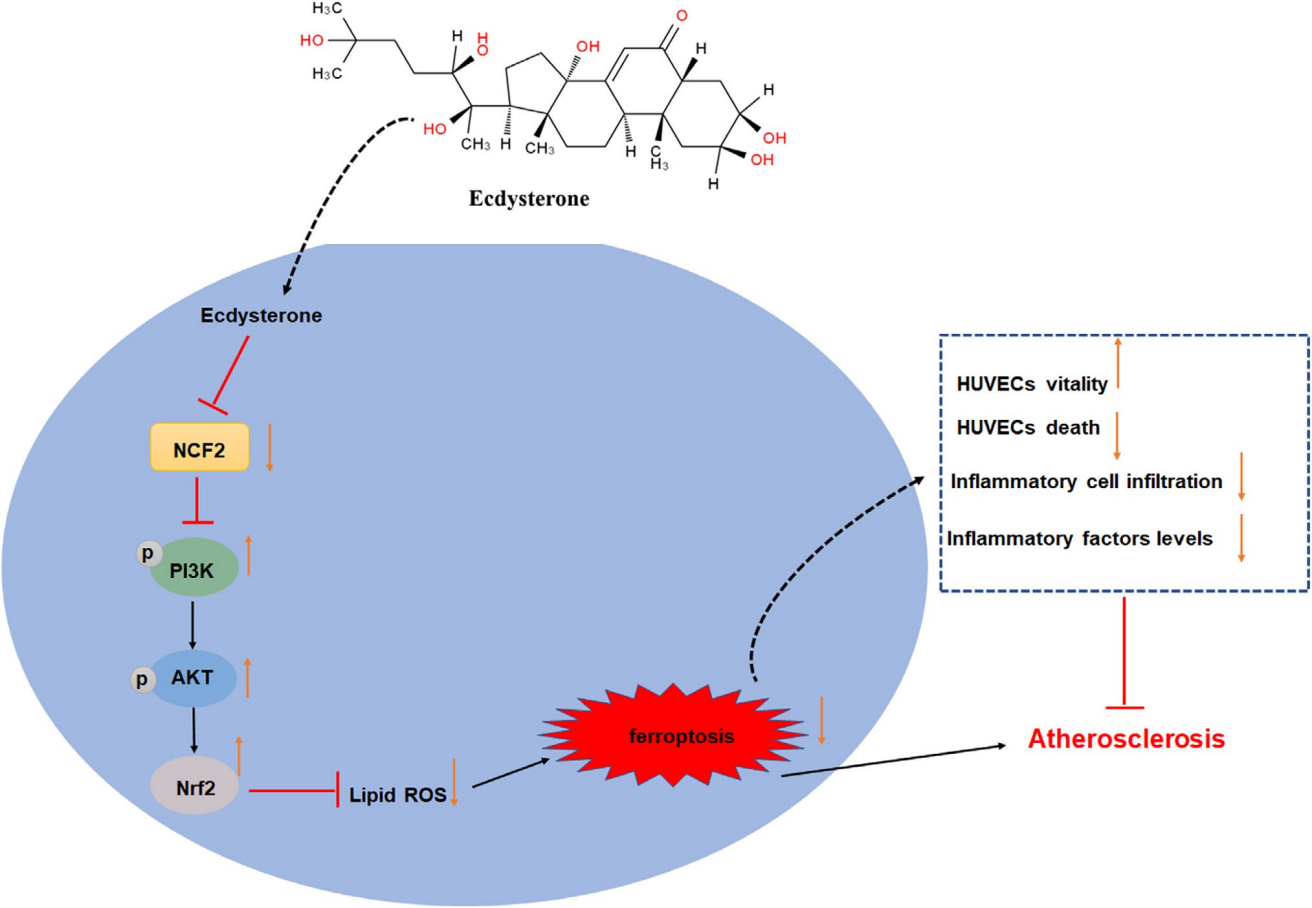
The pharmacological effect and mechanism of Ecdysterone in the prevention of endothelial ferroptosis and atherosclerosis.

In summary, Ecd downregulated NCF2 expression by binding to NCF2 and activated the PI3K/Akt/Nrf2 pathway, thereby inhibiting ox‐LDL‐treated HUVECs ferroptosis and alleviating HFD‐fed ApoE^−/−^ mice with AS (Figure 11). This study revealed the mitigation effect of Ecd on AS. It is worth noting that the administration of Ecd treatment may inhibit ferroptosis and inflammatory factor levels and improve vascular morphology and the degree of AS lesions. In addition, the protective effect of Ecd on AS was reflected by the inhibition of NCF2 and activation of the PI3K/Akt/Nrf2 signalling pathway. Although we have confirmed the mechanism of Ecd alleviating AS by inhibiting NCF2 and activating the PI3K/Akt/Nrf2 pathway whether Ecd can be used as a potential drug for the treatment of AS still needs to be verified in an expanded clinical population.

## Author Contributions

**Zhenyu Wang:** conceptualization (equal), validation (equal), writing – original draft (equal), writing – review and editing (equal). **Fengchao Wu:** conceptualization (equal), validation (equal), writing – review and editing (equal). **Ju Yan:** conceptualization (equal), validation (equal), writing – review and editing (equal). **Lei Liang:** data curation (equal), formal analysis (equal), validation (equal), writing – review and editing (equal). **Fengjun Chang:** data curation (equal), formal analysis (equal), visualization (equal), writing – review and editing (equal). **Mengya Dong:** visualization (equal), writing – original draft (equal), writing – review and editing (equal). **Jiayu Diao:** visualization (equal), writing – review and editing (equal). **Haoyu Wu:** funding acquisition (equal), writing – original draft (equal), writing – review and editing (equal).

## Ethics Statement

This study was approved by the Ethics Committee of the Shaanxi Provincial People's Hospital.

## Consent

All authors consent to publish this work.

## Conflicts of Interest

The authors declare no conflicts of interest.

## Supporting information


**Figure S1.** Ecdysterone inhibits Erastin‐induced ferroptosis and mitochondrial damage in HUVECs. HUVECs were treated with erastin (5 μM) for 8 h, and then treated with Ecd for 24 h. (A) MTT was used to detect the activity of HUVECs cells. (B) Determination of ferrous ion level by iron determination kit method. (C) MDA assay kit was used to detect MDA content. (D) DCFH‐DA fluorescent probe method was used to detect lipid ROS levels. (E) GPX4 activity was analysed using GPX4 assay kit. (F) Observation of mitochondrial morphology using transmission electron microscope. The data is expressed in means ± SD, *n* = 4; Student’s *t* test was used for comparison between the two groups, and one‐way analysis of variance was used for comparison between multiple groups; compared with control group *p < 0.05, compared with Erastin group #*p* < 0.05.
**Figure S2.** Atorvastatin attenuates HFD‐induced AS in ApoE^−/−^ mice. ApoE^−/−^ mice were fed a high‐fat diet for 10 weeks to establish the AS mouse model. During this period, ATO (10 mg/kg) was fed daily. (A) Oil red O staining was used to detect the degree of arterial plaque. (B) The levels of serum TC, TG, LDL and HDL were detected. (C) The secretion levels of inflammatory factors TNF‐α, IL‐6 and IL‐1β in serum were detected using ELISA. The data is expressed in means ± SD, *n* = 8. Student’s *t*‐test was used for comparison between the two groups, and one‐way analysis of variance was used for comparison between multiple groups; compared with WT group *p < 0.05, compared with AS group #*p* < 0.05.
**Figure S3.** Biosafety verification of Ecd in vivo. Two groups of C57BL/6 mice were intravenously injected with saline and Ecd (50 mg/kg) every 4 days for half a month to evaluate the in vivo safety of Ecd. (A) Body weight of mice. (B) The blood oxygen partial pressure and oxygen saturation were detected by IRMATRUPOINT blood gas analyser. (C) The levels of ALT and AST in serum. (D) The levels of BUN and Cr in serum. (E) The levels of inflammatory factors in serum. (F) Blood glucose levels of mice. (G) After the mouse were sacrificed, the organs were separated and the weights of liver, kidney and spleen were measured. The data is expressed in means ± SD, *n* = 8. Student’s *t*‐test was used for comparison between the two groups.

## Data Availability

The datasets generated in the current study are available from the corresponding authors upon reasonable request.
