# Supplementary material for: Ecdysterone Alleviates Atherosclerosis by Inhibiting NCF2 and Inhibiting Ferroptosis Mediated by the PI3K/Akt/Nrf2 Pathway
Source: J Cell Mol Med. 2025 Mar 5;29(5):e70446. doi: 10.1111/jcmm.70446 (PMC11882393; doi:10.1111/jcmm.70446)
Supplement: Supplementary file 1 — Figure S1. Ecdysterone inhibits Erastin‐induced ferroptosis and mitochondrial damage in HUVECs. HUVECs were treated with erastin (5 μM) for 8 h, and then treated with Ecd for 24 h. (A) MTT was used to detect the activity of HUVECs cells. (B) Determination of ferrous ion level by iron determination kit method. (C) MDA assay kit was used to detect MDA content. (D) DCFH‐DA fluorescent probe method was used to detect lipid ROS levels. (E) GPX4 activity was analysed using GPX4 assay kit. (F) Observation of mitochondrial morphology using transmission electron microscope. The data is expressed in means ± SD, n = 4; Student’s t test was used for comparison between the two groups, and one‐way analysis of variance was used for comparison between multiple groups; compared with control group *p < 0.05, compared with Erastin group #p < 0.05. Figure S2. Atorvastatin attenuates HFD‐induced AS in ApoE−/− mice. ApoE−/− mice were fed a high‐fat diet for 10 weeks to establish the AS mouse model. During this period, ATO (10 mg/kg) was fed daily. (A) Oil red O staining was used to detect the degree of arterial plaque. (B) The levels of serum TC, TG, LDL and HDL were detected. (C) The secretion levels of inflammatory factors TNF‐α, IL‐6 and IL‐1β in serum were detected using ELISA. The data is expressed in means ± SD, n = 8. Student’s t‐test was used for comparison between the two groups, and one‐way analysis of variance was used for comparison between multiple groups; compared with WT group *p < 0.05, compared with AS group #p < 0.05. Figure S3. Biosafety verification of Ecd in vivo. Two groups of C57BL/6 mice were intravenously injected with saline and Ecd (50 mg/kg) every 4 days for half a month to evaluate the in vivo safety of Ecd. (A) Body weight of mice. (B) The blood oxygen partial pressure and oxygen saturation were detected by IRMATRUPOINT blood gas analyser. (C) The levels of ALT and AST in serum. (D) The levels of BUN and Cr in serum. (E) The levels of inflammatory factors in [file JCMM-29-e70446-s001.docx]

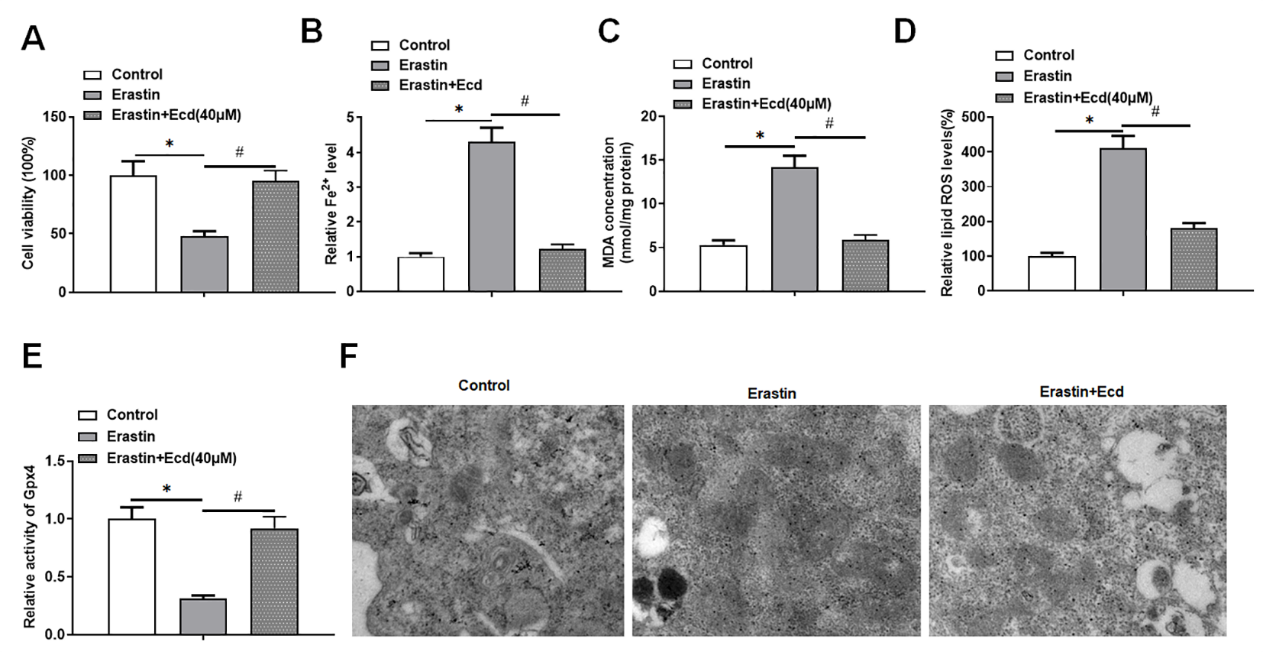


**Supplementary Fig.1 Ecdysterone inhibits Erastin-induced ferroptosis and mitochondrial damage in HUVECs**

HUVECs were treated with erastin (5 μM) for 8 hours, and then treated with Ecd for 24 hours. A. MTT was used to detect the activity of HUVECs cells. B. Determination of ferrous ion level by iron determination kit method. C. MDA assay kit was used to detect MDA content. D. DCFH-DA fluorescent probe method was used to detect lipid ROS levels. E. GPX4 activity was analyzed using GPX4 assay kit. F. Observation of mitochondrial morphology using transmission electron microscope. The data is expressed in means ± SD, n = 4; Student’s t test was used for comparison between the two groups, and one-way analysis of variance was used for comparison between multiple groups; compared with control group **P* < 0.05, compared with Erastin group #*P* < 0.05.

**
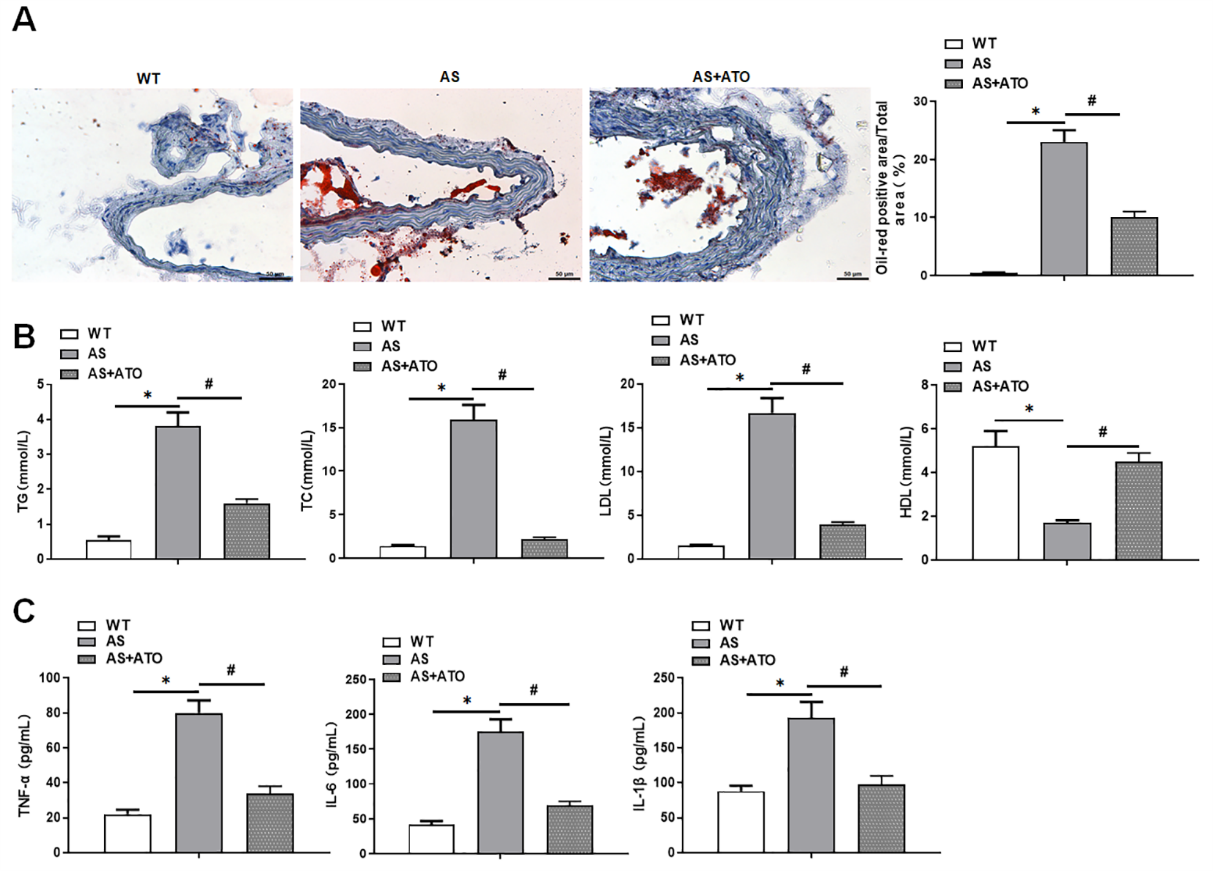
**

**Supplementary Fig.2 Atorvastatin attenuates HFD-induced AS in ApoE^-/-^ mice**

ApoE^−/−^ mice were fed a high-fat diet for 10 weeks to establish the AS mouse model. During this period, ATO (10 mg/kg) was fed daily. A. Oil red O staining was used to detect the degree of arterial plaque. B. The levels of serum TC, TG, LDL and HDL were detected. C. The secretion levels of inflammatory factors TNF-α, IL-6 and IL-1β in serum were detected using ELISA. The data is expressed in means ± SD, n = 8. Student’s t-test was used for comparison between the two groups, and one-way analysis of variance was used for comparison between multiple groups; compared with WT group **P* < 0.05, compared with AS group #*P* < 0.05.

**
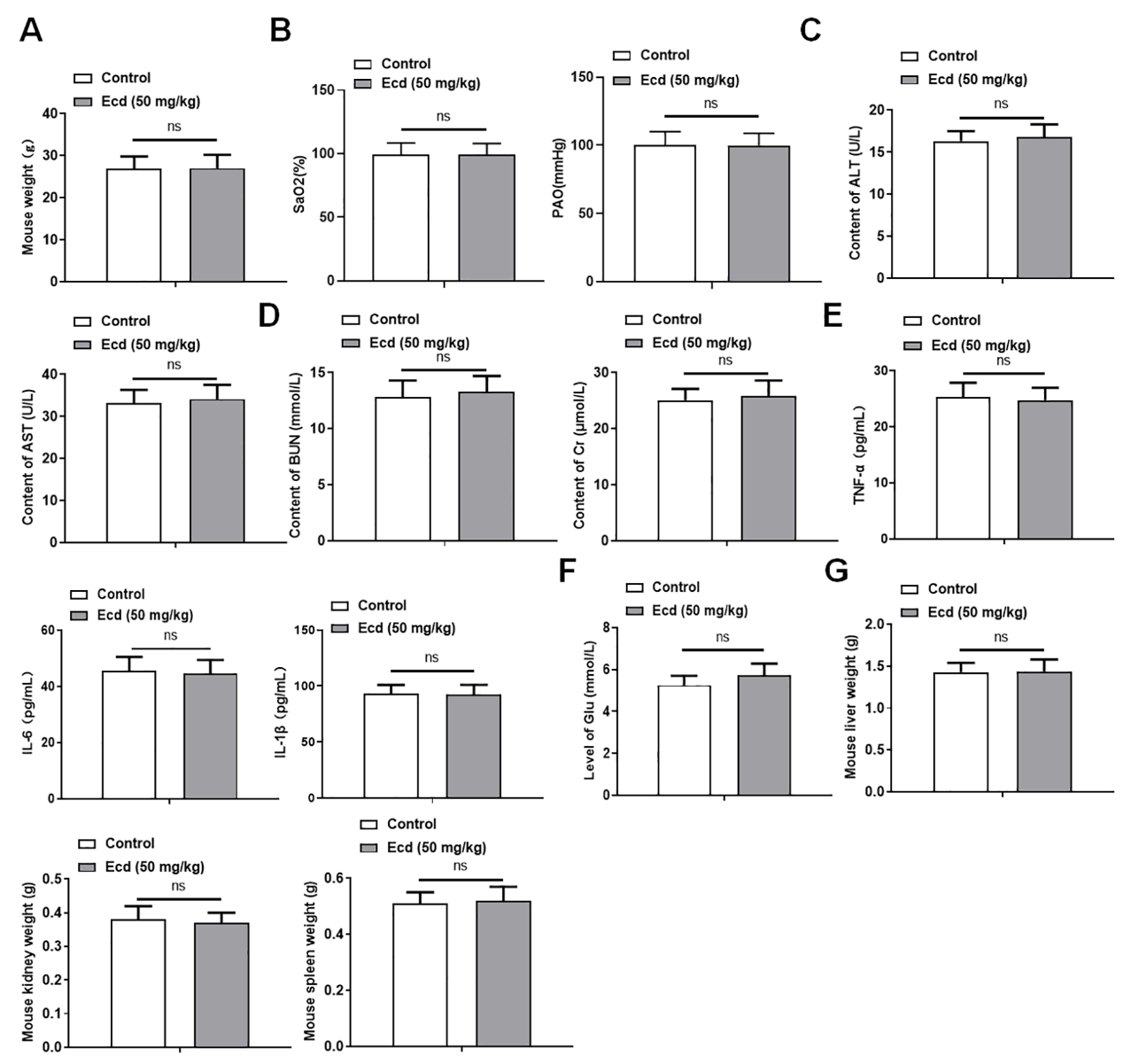
**

**Supplementary Fig.3 Biosafety verification of Ecd *in vivo***

Two groups of C57BL/6 mice were intravenously injected with saline and Ecd (50 mg/kg) every 4 days for half a month to evaluate the in vivo safety of Ecd. A. Body weight of mice. B. The blood oxygen partial pressure and oxygen saturation were detected by IRMATRUPOINT blood gas analyzer. C. The levels of ALT and AST in serum. D. The levels of BUN and Cr in serum. E. The levels of inflammatory factors in serum. F. Blood glucose levels of mice. G. After the mouse were sacrificed, the organs were separated and the weights of liver, kidney and spleen were measured. The data is expressed in means ± SD, n = 8. Student’s t-test was used for comparison between the two groups.
